# Co-regulation of microglial subgroups in Alzheimer’s amyloid pathology: Implications for diagnosis and drug development

**DOI:** 10.1371/journal.pone.0337741

**Published:** 2025-12-05

**Authors:** Yu Zhou, Yukuan Huang, Yangchang Fan, Feng Xue

**Affiliations:** Hwamei College of Life and Health Sciences, Zhejiang Wanli University, Ningbo, Zhejiang, China; UCLA Doheny Eye Institute, IRAN, ISLAMIC REPUBLIC OF

## Abstract

Alzheimer’s Disease (AD) is a progressive neurodegenerative disorder and the leading cause of dementia. Neuroinflammation drives AD progression and therefore represents a promising target for diagnosis and therapy. In early AD, microglia polarize into pro-inflammatory and anti-inflammatory cellular subgroups that mediate the initial immune response, yet the regulatory relationships between these microglial subgroups remain poorly understood. In this study, we investigated the interplay between pro- and anti-inflammatory microglial subgroups from multiple perspectives. Comparative transcriptomics and bioinformatics analyses implicated the Trem2 signaling pathway in an anti-inflammatory microglial subgroup. Fluorescence-activated cell sorting (FACS) and gene regulation analysis indicated that microglial subgrouping and microgliosis preceded cytokine upregulation during early amyloid pathology. Further immunoassays revealed that anti-inflammatory Neurodegeneration-Related Modules and pro-inflammatory microglial subgroups, Interferon-Related Modules and LPS-Related Modules, were co-regulated by shared upstream pro-inflammatory regulators. Such co-regulation of heterogeneous microglial subgroups may balance microglial activation and promote the development of AD chronic neuroinflammation. In summary, our study uncovered previously overlooked co-regulation of microglial subgroups in AD and provides a systems biology framework that may inform improved diagnostic markers and immunotherapeutic strategies.

## Introduction

As a progressive neurodegenerative disorder, Alzheimer’s Disease (AD) is the most common cause of dementia [1]. It is characterized by persistent and progressive memory loss, cognitive impairment, and personality changes. The hallmark pathological features of AD include amyloid plaques formed by extracellular accumulation of amyloid beta proteins (Aβ), intracellular neurofibrillary tangles composed of hyperphosphorylated tau, and progressively evolving chronic neuroinflammation [2]. Microglia are the principal innate immune myeloid cells of the central nervous system (CNS), acting as tissue-resident phagocytes in both infection and neurodegeneration [3]. In the healthy CNS, homeostatic microglia continuously survey the microenvironment, sculpt developing neuron circuit and synapses, and clear cellular debris. In pathological conditions, microglia respond to homeostatic perturbations and transition into an “activated” state. Microglial activation is a precisely regulated process that encompasses signaling pathway activation, morphological transformation, changes in surface markers, cytokine secretion, and cellular proliferation [4].

Microglial activation in AD plays a dual role. On one hand, microglia contribute to protection by phagocytosing and clearing toxic Aβ species. On the other hand, microglia can exacerbate pathology by mediating synapse loss in an immune-dependent manner and by promoting neuroinflammation that aggravates neuronal injury and cognitive deficits. Early *in vitro* work described canonical “M1-like” pro-inflammatory activation via Toll-like receptors and MyD88-NF-κB and JAK-STAT signaling [5,6], and “M2-like” anti-inflammatory activation via the IL-10-IL-10R axis [7]. More recent single-cell RNAseq (scRNAseq) and single-nucleus RNAseq (snRNAseq) transcriptomics studies have revealed multiple subgroups of activated microglia in AD mouse models and in human postmortem samples [8,9]. These studies further suggested that heterogeneous microglia subgroups established a dynamic balance between pro-inflammatory and anti-inflammatory microglial activation [10,11]. Nevertheless, the detailed regulatory networks governing these microglia subgroups remain incompletely defined.

In this study, we performed a comparative transcriptomics analysis between Major Depressive Disorder (MDD) acute neuroinflammation and AD chronic neuroinflammation to evaluate the role of Trem2-mediated Neurodegeneration-Related Modules (NEUD-MDL), often referred to as Disease-Associated Microglia (DAM), in anti-inflammatory microglial activation in AD. Complementary pharmacological activation and inhibition assays in primary microglial cultures demonstrated that several pro-inflammatory signaling pathways co-regulated both pro-inflammatory and anti-inflammatory microglial activation in AD. Notably, the top-ranked predicted upstream signaling pathways, TNF and NF-κB, appeared to modulate microglial activation in an age-dependent manner. Collectively, these findings improve our understanding of microglial activation in AD chronic neuroinflammation and provide a systems-level framework that may inform development of diagnostic markers and immunotherapeutic strategies for neurodegenerative diseases.

## Materials and methods

### Animal models

All animal experiments were reviewed and approved by the Institutional Animal Care and Use Committee of Zhejiang Wanli University. The C57BL/6 Wild-type (WT) mouse models and the 5×FAD mouse models [B6.Cg-Tg(APPSwFlLon,PSEN1*M146L*L286V)6799Vas/Mmjax] were obtained from Shanghai Model Organisms Center, Inc. (SMOC). The genotypes of 5×FAD mouse models were verified using PCR and/or Aβ plaque staining. The Lipopolysaccharide (LPS)-induced MDD mouse models (MDD-LPS24h) were established according to a previously described protocol [12]. All animals were euthanized in accordance with the American Veterinary Medical Association (AVMA) Guidelines. Briefly, mouse models were humanely euthanized via CO₂ overdose followed by cervical dislocation. Death was confirmed by the absence of respiration and corneal reflex.

### RNAseq and data processing

For transcriptomics analysis, 3 mice per group (model or control) were randomly selected and euthanized. The hippocampus (HPC) of each mouse was dissected, immediately homogenized in TRIzol™ Reagent (Invitrogen, 15596018), snap-frozen in liquid nitrogen, and stored at -80°C until processing. Transcriptomics library preparation and RNAseq were finished by Berry Genomics Co., Ltd. (Beijing, China) on an Illumina NovaSeq 6000 system following the standard protocols of the manufacturer (Illumina, USA). Raw FASTQ files were processed using an in-house pipeline. Reads were aligned to the murine genome (mm39) and gene annotation GENCODE vm31 using the STAR aligner (v2.7.10b) with the following parameters: --outFilterType BySJout --outFilterMultimapNmax20 --outFilterMismatchNmax999 --outFilterMismatchNoverReadLmax0.04 --alignIntronMin20 --alignIntronMax1000000 --alignMatesGapMax 1000000 --alignSJoverhangMin8 --alignSJDBoverhangMin1. Gene-level expression was quantified using RSEM (v1.3.3) with the default parameters. Differential expression analysis was performed in R (v4.3.2) using the DESeq2 package (v1.33.0) with default parameters. Gene identifiers were mapped to GENCODE vM31 symbols using BioMart; where direct mappings were unavailable, external_synonym fields were used to retrieve alternative identifiers. Heatmaps of Z-scaled normalized transcripts per million (TPM) values, significance levels and log fold change on genes of subtypes were produced by the ComplexHeatmap (v2.14.0) library in R (v4.3.2). All computations were conducted in the HUAWEI Cloud virtual server computing environment [13]. The significant “differentially expressed genes (DEGs)” (adj. *p* < 0.05) were selected for downstream bioinformatics analysis.

### Bioinformatics data mining

In the comparative transcriptomics analysis, Mechanistic Network of Upstream Regulators, Regulator Effect Network, and Causal Network were reconstructed using Ingenuity Pathways Analysis (IPA, QIAGEN) with default statistical settings [10,14]. The predicted Upstream Cytokines, Transcription factors, and Receptors of the microglial subgroup markers were extracted for comparative analysis. Marker gene lists of the major microglial subgroups [15] and Interferon-Stimulated Genes (ISGs) lists [16] were obtained from the respective references. The Gene Set Enrichment Analysis (GSEA) was performed with the R package clusterProfiler (v.4.2.2). Multiple testing corrections, including Bonferroni, false discovery rate (FDR), and Benjamini–Hochberg methods, were applied, with pathways considered significant at an adjusted *p* < 0.05. The network robustness was assessed by bootstrap resampling (n = [1000] iterations), retaining edges only if reproducible in >95% of runs. The pathway predictions were derived from consensus databases (IPA and Reactome) and were cross-validated by leave-one-out analysis. For the meta-analysis, the scRNAseq datasets were acquired from Tabula Muris (https://tabula-muris.ds.czbiohub.org/) [17] and ZEBRA (https://www.ccb.uni-saarland.de/zebra) [18]. The gene regulation data from human postmortem samples and mouse models were acquired from The Myeloid Landscape 2 (http://research-pub.gene.com/BrainMyeloidLandscape/BrainMyeloidLandscape2/) [15]. The microglial scRNAseq/ snRNAseq datasets from individual human postmortem samples and mouse models were acquired from scREAD: A Single-cell RNA-Seq Database for Alzheimer’s Disease (https://bmbls.bmi.osumc.edu/scread/) [19]. The known protein-protein interactions and transcription factor-target gene relationships were retrieved from the OmniPath database [20,21]. The graphical network representations were generated using Cytoscape (3.9.1) [22].

### Fluorescence-activated cell sorting (FACS)

For the FACS-based purification of CD11b^+^ microglia, 6 mice from each model and control group were randomly selected for euthanasia. The experiments were performed according to previously described methods with minor modifications [23]. Briefly, the whole cortex was dissected and washed twice with ice-cold Dulbecco’s phosphate-buffered saline (DPBS, Gibco, 14190144). The brain tissue was dissociated with Accutase (Innovative Cell Technologies, NC9839010). The debris and myelin were removed with Debris Removal Solution (Miltenyi Biotec, 130-109-398). The cell suspension was washed with ice-cold DPBS and centrifuged at 300×g for 10 min at 4 °C. The cell pellet was resuspended, fixed in 50% ice-cold ethanol for 15 min, and centrifuged at 500×g for 10 min at 4 °C. The resulting pellets were resuspended in ice-cold DPBS and subjected to immunostaining at 4 °C for 20 min. The following antibodies and reagents were used: Alexa Fluor 488-conjugated anti-NeuN (Millipore, MAB377X, 1:1000), PE-conjugated anti-GFAP (BD Biosciences, 561483, 1:50), APC-conjugated anti-CD11b (BD Biosciences, 561690, 1:250), and DAPI (Invitrogen, D21490, 5ng/mL). After staining, the cells were washed twice and resuspended in ice-cold DPBS containing 0.5% BSA. Cell sorting was performed on a BD FACSAria™ Fusion instrument. The DAPI^+^/singlet cells were selected as the parent population to exclude cell debris, and the CD11b^+^/NeuN^-^/GFAP^-^ cells were sorted directly into TRIzol (Invitrogen, 15596018).

### Magnetic-activated cell sorting (MACS)

The primary microglia (PMG) were isolated from mouse models using the CD11b (Microglia) MicroBeads (Miltenyi Biotec, 130-093-634) following the manufacturer’s protocol. Briefly, the meninges and blood vessels of the randomly selected adult mouse brains were carefully removed under a dissection microscope. The whole cortex was then minced and dissociated with Accutase (Innovative Cell Technologies, 00-4555-56) treatment for 15 min at 4°C. Cellular debris was removed using Debris Removal Solution (Miltenyi Biotec, 130-109-398), and PMG were subsequently purified with CD11b (Microglia) MicroBeads according to the manufacturer’s protocol. The purified PMG were immediately cultured in Dulbecco’s Modified Eagle Medium (DMEM) (Gibco, 11965092) supplemented with 10% heat-inactivated FBS (Gibco, 16140089) and 1% Penicillin-Streptomycin (PS) (Gibco, 15140122) at 37°C in a humidified incubator containing 5% CO_2_. The cells were seeded onto 24-well plates (Corning, CLS3524-100EA) at a density of 20,000 cells/cm^2^. All tissue and cell experiments were performed on ice or at 4 °C using endotoxin-free solutions to minimize contamination and prevent unintended activation of gene expression.

### Pharmacological activation and inhibition assays

Prior to the pharmacological activation and inhibition assays, the primary microglia purified from the Wild-type mouse models (WT-PMG) or the 5×FAD mouse models (FAD-PMG) were cultured *in vitro* for 18 h to reach a steady state. Subsequently, the immune cytokines and activators, including Ifna, Ifng, Tnf, and *E. coli* LPS, or the immune inhibitors, including TNF-alpha Antagonist III, R-7050 (TA), and NF-κB inhibitor Prostaglandin E2, EP1, EP2, EP3, and EP4 ligand (PGE2), were added into the cultured cells, respectively. The final concentrations were 10 μg/mL for cytokines, 10 ng/mL for *E. coli* LPS, and 10 μM for the inhibitors (S1 Table). Immediately after treatment, the cultured cells were gently mixed by vortexing and incubated for an additional 8 h. Following the 8-hour induction or inhibition, the cultured cells were quickly lysed in TRIzol (Invitrogen, 15596018) and stored at -80°C until cDNA synthesis. For each treatment, 3 biological replicates (wells) were prepared, and the experiments were biologically repeated in at least 2 independent batches of MACS isolation and cell culture.

### cDNA synthesis and quantitative PCR (qPCR)

The brain tissue from mouse models was immersed in TRIzol (Invitrogen, 15596018), frozen in liquid nitrogen, and homogenized using RNase-/DNase-free pestles in 1.5 mL tubes. For cellular samples, 10,000 FACS-purified microglial cells or PMG cultures, were fully lysed in TRIzol (Invitrogen, 15596018). Total RNA was purified by ZYMO Direct-zol RNA Microprep Kits (ZYMO, R2063). The first-strand cDNA was synthesized with PrimeScript RT Master Mix (TaKaRa, RR036A) using random primers (TaKaRa, 3801). For the lowly expressed transcripts, the cDNAs were pre-amplified by the PerfeCTa PreAmp SuperMix (Quantabio, 95146-040) following the manufacturer’s protocol. The qPCR reactions were performed on an Applied Biosystems StepOnePlus Real-Time PCR System using PowerUp™ SYBR™ Green Master Mix (Applied Biosystems, A25742) with ROX reference dye (Invitrogen, 12223012). All RNA experiments were performed under RNase-/DNase-free conditions to minimize RNA degradation and DNA contamination. The relative quantitation data were acquired with the 2^−ΔΔCT method, using mouse *Gapdh* as the internal control. Then, the regulation of the target genes in 5×FAD microglia was determined by calculating fold change relative to NonTg control samples. The oligo primers that were used in cDNA pre-amplification and qPCR are listed in S2 Table. The bar figures and statistics were generated by the GraphPad Prism 9 software, and the heatmaps of significantly regulated genes (*p* < 0.05) were produced by the ComplexHeatmap (v2.14.0) library in R (v4.3.2).

### Western blotting

For protein analysis, the HPC or the prefrontal cortex (PFC) tissues from mouse models were rapidly dissected, immediately frozen in liquid nitrogen, and stored at -80°C until use. The frozen tissues were homogenized in ice-cold lysis buffer (Beyotime, P0013C) supplemented with PMSF (Beyotime, ST506), EDTA-free protease inhibitor (Beyotime, P1005), and phosphatase inhibitor (Beyotime, P1081). The homogenates were centrifuged at 12,000×g for 10 min at 4 °C, and the protein concentration in the supernatants was determined using the BCA Protein Assay Kit (Beyotime, P0012). Equal amounts of protein were separated on 12% SDS-PAGE SurePAGE™ (Genscript, M00669) and transferred onto polyvinylidene fluoride membranes (Merck, 3010040001). Then the membranes were blocked and incubated overnight at 4 °C with mouse primary antibodies GFAP (1:5000, Proteintech, 16825-1-AP), AIF1 (IBA1) (1:1000, Cell Signaling Technology, 17198), TSPO (1:20000, Abcam, ab109497), and β-Actin (1:5000, Proteintech, 81115-1-RR). After washing, the membranes were incubated with secondary anti-rabbit HRP-conjugated antibodies (1:10000, Sangon, D110058-0001) and visualized using the enhanced chemiluminescent HRP substrate (Beyotime, P0018FS). The protein band intensities were captured using the Tanon 5200 Gel Imaging System (Tanon, China) and analyzed with ImageJ 1.54i software (National Institutes of Health, USA). The bar figures and statistics were generated by the GraphPad Prism 9 software.

### Immunofluorescence staining

The mouse brain tissue was immediately fixed in 4% paraformaldehyde (Absin, abs9179) overnight at 4 °C. The fixed brains were subsequently dehydrated in a graded sucrose solution (10%-30% sucrose Beyotime, ST1670-250g) prepared in PBS and embedded in Tissue freezing medium OCT (Sakrua, USA) before storage at -80 °C. Coronal sections were cut using a CM1950 cryostat (Leica, USA) into 25-µm slices. The slices were blocked with serum (Absin, abs946) for 2 h at room temperature, followed by incubation with the primary antibodies GFAP (1:1000, Cell Signaling Technology, 12389) and AIF1 (IBA1) (1:1000, WAKO, 019-19741) overnight at 4 °C. After washing, the slices were incubated with secondary antibodies (1:1000, Proteintech, srbAF488-1) and DAPI (Beyotime, C1002). Finally, the samples were mounted using Fluoromount™ Aqueous Mounting Medium (Sigma, F4680). The fluorescence images were acquired using either an Echo Revolve Fluorescence Microscope (BICO, USA) or a STELLARIS 5 Cryo Confocal Light Microscope (Leica, USA). The quantification of fluorescence intensity and cell counting in the hippocampus, including the CA1 and dentate gyrus, were performed using ImageJ 1.54i software (National Institutes of Health, USA). The fluorescence density measurements were conducted in a single-blind manner. The bar figures and statistics were generated by the GraphPad Prism 9 software.

### Property prediction of chemical compounds

The molecular properties of the anti-inflammatory chemical compounds used or discussed in this study, including the p38-MAPK inhibitors Neflamapimod and Losmapimod, the NF-κB inhibitor NE3107 (HE3286) and Prostaglandin E2, EP1, EP2, EP3, and EP4 ligand (PGE2), the CSF1R inhibitor Masitinib, and the TNF-alpha Antagonist III, R-7050 (TA), were predicted using the HUAWEI Cloud Pangu Drug Molecular Model (http://www.pangu-drug.com/) [13]. In brief, the canonical SMILES of the chemical compounds were entered into the PanGu Fingerprint system to predict the basic molecular characteristics, including BACE, BBBP, Tox21, ToxCast, FreeSolv, ESOL, and QM7. The prediction data on distribution, absorption, and toxicity of the chemical compounds were then compiled and compared in tabular form.

### Statistical analysis

All the mRNA and protein quantification data, excluding the RNAseq data, were analyzed using the GraphPad Prism 9 software and were presented as mean ± SEM. The differences among treatments and genotypes were assessed by one-way or two-way analysis of variance (ANOVA), followed by Tukey’s post hoc test. *p* < 0.05 was considered statistically significant.

## Results

### Human AD risk factors were related to the activation of immune pathways

**Fig 1:**
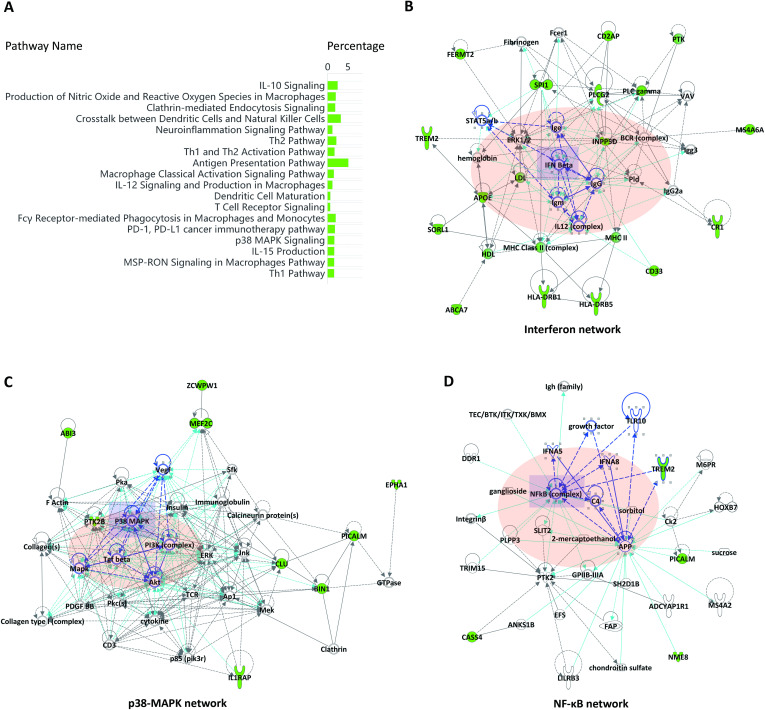
Major human AD risk factors converge on immune regulation networks. (A) The pathway enrichment (IPA-Cellular Immune Response Pathways) for major AD risk factors (full gene lists in S3 Table). (B) The Top 1 IPA network: Type I interferon–related signaling pathways (IFNB1-STAT5A/B, IL-12, MHC II, IgG). (C) The Top 2 IPA network: Growth-factor signaling pathways (TGF-β, PI3K–AKT, p38-MAPK). (D) The Top 3 IPA network: APP-associated immune signaling pathways (APP, TLR10, IFNA, NF-κB). Multiple testing corrections were applied in pathway analysis, and the pathways with adjusted p < 0.05 were considered significant. Network robustness was assessed by bootstrap resampling (n = [1000]), and the edges were retained if present in >95% of runs.

The major AD risk factors, including ABI3, ABCA7, CD2AP, CD33, CASS4, EPHA1, NME8, SPI1, APOE, BIN1, CLU, CR1, FERMT2, INPP5D, IL1RAP, HLA-DRB1, HLA-DRB5, MS4A6A, MEF2C, PICALM, PLCG2, PTK2B, SORL1, TREM2, and ZCWPW1, have been identified through Genome-Wide Association Studies (GWAS). Several of these genes have been validated as immune-related in animal models [24–26]. However, the immune regulations of these AD risk factors remain largely unknown. In this study, the IPA Canonical Pathway analysis revealed that these AD risk factors were broadly involved in multiple immune activation pathways (Fig 1A). The IPA Network analysis further reconstructed several immune regulation networks. The TOP 1 IPA network was associated with the Interferon signaling pathway, with IFN I, IL12 complex, MHC II, IgG, and IgM identified as upstream regulators (Fig 1B). The TOP 2 IPA network was linked to the P38-MAPK signaling pathway, with TGF-beta, PI3K-AKT, and P38-MAPK as upstream regulators (Fig 1C). The TOP 3 IPA network involved the NF-κB immune pathway, with APP, TLR10, IFNA, and NF-κB as upstream regulators (Fig 1D). Collectively, these network predictions suggested that the AD risk factors may play roles in the gene regulation of immune activation.

The IPA Upstream Regulator analysis further revealed that numerous pro-inflammatory and anti-inflammatory signaling pathways may regulate the expression of AD risk factors. For instance, TGFB1 positively regulated APOE and MEF2C, whereas LPS and IL6 negatively regulated APOE, CD33, MEF2C, and TREM2. TGFB1 acts as a protective growth factor that maintains microglia homeostasis, while LPS and IL6 are key immune mediators driving microglial pro-inflammatory activation and neuroinflammation. Moreover, the IPA Causal Network analysis predicted a series of master regulators that may directly or indirectly control the expression of the major AD risk factors (S3 Table). For example, IFNGR, Notch, PI3K, STAT5A/B, TLR2/3/4/9, IGF2, MAPK13, miR-155, IL9R, and CSF2RA were identified as potential negative Master Regulators, whereas TH2 Cytokines, CSF, JAK1/2, TBK1, and B2M were predicted to be positive Master Regulators. These upstream predictions suggested that the immune signaling may modulate the expression of the AD risk factors under both physiological and pathological conditions.

Among these genes, the triggering receptor expressed on myeloid cell 2 (TREM2) gene has recently been identified as an AD risk factor through GWAS [27]. TREM2 encodes a transmembrane immune receptor involved in phagocytosis and inflammatory response [28]. Lipids (neutral and anionic) [29], APOE protein [30], Aβ protein [31], and CD33 [32] had been verified as upstream regulators of the TREM2 signaling pathway. In the present IPA analysis, the APOE-TREM2 axis was found to be co-regulated by the pro-inflammatory signaling pathways (e.g., CXCL16, TNFSF8, and TLR2/3/4/9) and the anti-inflammatory TH2 cytokines (S3 Table). These Causal Network predictions suggested that TREM2 signaling functioned not only as an upstream signaling pathway but may also participate in downstream feedback regulation [10].

In addition, the Type I interferon (IFN I) and growth factors (TGFb, CSFs, VEGFC, and IGF2) were predicted to participate in the regulation of the AD risk factors (S3 Table). Notably, the risk factor MEF2C was found to be regulated by both TGFb and IFN I signaling pathways, consistent with previous findings that it was regulated in activated microglia during aging and in AD models [26,33]. In summary, these analyses indicated that the key regulation networks of the AD risk factors corresponded closely to microglial activation and subgroups in AD chronic neuroinflammation [34].

### AD microglial subgroups markers formed networks of immune activation

Microglial activation has been shown to mediate early neuroinflammation in AD. This process involves both pro-inflammatory and anti-inflammatory signaling pathways, as well as the programmed differentiation of microglial subgroups. Recent scRNAseq studies have identified several novel microglial subgroups and their molecular markers in AD mouse models and postmortem human samples. A major microglial subgroup, Disease-Associated Microglia (DAM), was first identified in the 5 × FAD mouse model [8]. The DAM represented the predominant microglial phenotype, as it was defined by semi-supervised clustering with pre-set immune cell outgroups (CD45^+^ T/B cells). A subsequent meta-analysis further delineated Neurodegeneration-Related Modules (NEUD-MDL), Interferon-Related Modules (IFN-MDL), LPS-Related Modules (LPS-MDL), and Proliferation-Related Modules (PROL-MDL) [15]. Other scRNAseq hierarchical clustering studies have defined H1M/H2M, TRE, IRM, ARM, and CPM microglial subgroups [35], as well as Proliferative-region-associated microglia (PAM) [36]. Several DAM mRNA markers, including Apoe and Trem2, have been validated at the protein level by proteomics research [37]. Notably, the DAM subgroup corresponded to the NEUD-MDL and the previously described MGnD phenotype [15,38]. Moreover, these microglial subgroups also partially aligned with the above-mentioned immune regulation networks of the major AD risk factors [34].

**Fig 2:**
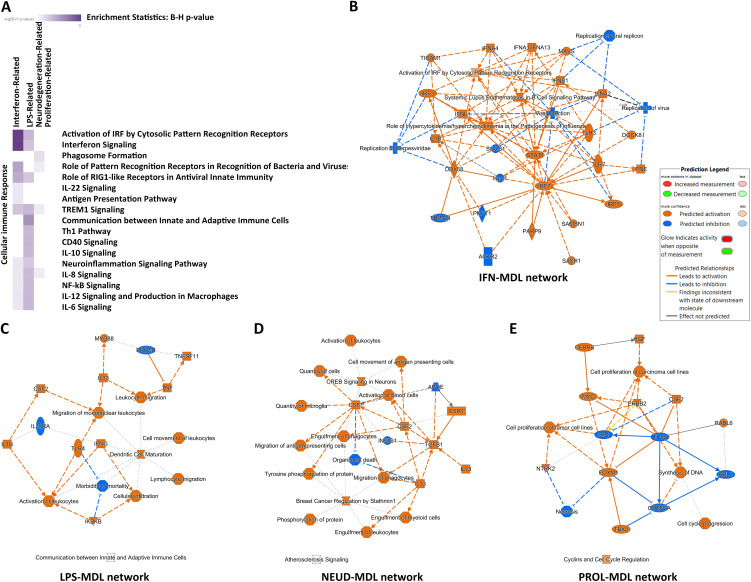
Pathway and network analysis of microglial subgroups. (A) The IPA Canonical Pathway analysis of the microglial subgroups: The IFN-MDL and LPS-MDL clustered as pro-inflammatory microglial subgroups. (B) The IPA Graphic Summary of IFN-MDL network: Antiviral immune responses. (C) The IPA Graphic Summary of LPS-MDL network: Pro-inflammatory receptor and cytokine signaling pathways. (D) The IPA Graphic Summary of NEUD-MDL network: Growth-factor signaling pathways. (E) The IPA Graphic Summary of PROL-MDL network: TP53-related cell-cycle pathways. Multiple testing corrections were applied in pathway analysis, and the pathways with adjusted p < 0.05 were considered significant. Network robustness was assessed by bootstrap resampling (n = [1000]), and the edges were retained if present in >95% of runs. The IPA Prediction Legend was included in the figure.

To investigate the immune regulations of the microglial subgroups, we performed Canonical Pathway analysis, Upstream Regulator analysis, and Causal Network analysis using the major mRNA markers of the microglial subgroups. Comparison among the subgroups revealed that the NEUD-MDL (Trem2-DAM) mediated anti-inflammatory microglial activation [10], whereas the IFN-MDL and the LPS-MDL caused pro-inflammatory microglial activation (Fig 2A). Importantly, the “Neuroinflammation Signaling Pathway” was significantly enriched in both IFN-MDL and LPS-MDL. However, the previously published scRNAseq clustering data showed that these two pro-inflammatory subgroups contained fewer cells compared to the anti-inflammatory NEUD-MDL [15].

Within the IFN-MDL, the Canonical Pathway “Activation of IRF by Cytosolic Pattern Recognition Receptors” was the top-enriched immune signaling pathway. This suggested that this microglial subgroup may be induced by intracellular stress factors (Fig 2A). Consistently, the Causal Network analysis revealed that the “STING-TRAF3-TBK1” and “IRF3-IRF7” antiviral signaling networks were more prominent than the downstream “IFNAR” signaling networks (Table 1). The “STING-TRAF3-TBK1” pathway senses cytosolic double-stranded DNA fragments as Damage-Associated Molecular Patterns (DAMPs) and/or Pathogen-Associated Molecular Patterns (PAMPs), and then initiates Ifn I synthesis and the downstream IFNAR signaling cascade.

**Table 1:** Master Regulators of the Top 10 Causal Networks of the microglial subgroups.

IFN-MDL	LPS-MDL	NEUD-MDL	PROL-MDL
Sting-Traf3-Tbk1 (4.79)	Mlkl (4.14)	Prkd1 (3.20)	Igfbp2 (3.46)
Irf3-Irf7 (4.79)	Rhebl1 (4.02)	Tnf (3.16)	Csf (3.16)
Irf7 (4.58)	NF-κB (complex) (3.90)	Th1 cytokine (3.05)	Egfr ligand (3.16)
Irf3 (4.58)	Creb-NF-κB (3.77)	Ccr1 (2.84)	Cdc14B (3.16)
LPS (4.35)	LPS (3.74)	Nfat (family) (2.64)	Cdc14A (3.16)
Ifnar (4.24)	Th1 cytokine (3.74)	Smad2/3/4 (2.50)	S100A6 (3.16)
Ifnl4 (4.24)	Clock (3.57)	Ap1 (2.33)	Pax3 (3.16)
Parp9 (4.24)	Irf3 (3.46)	Smad5 (2.30)	Ski (3.16)
cGAS (4.24)	Scube3 (3.35)	Lifr (2.13)	Mdm4 (3.16)
Ifna (4.24)	Hyaluronidase (3.31)	Chemokine (2.12)	Rala (3.16)

Note: The Activation Z-scores were listed in the brackets.

Interestingly, both the “Activation of IRF by Cytosolic Pattern Recognition Receptors” and the “Interferon Signaling” were also significantly enriched in the LPS-MDL (Fig 2A), suggesting that the IFN I signaling may co-regulate the IFN-MDL and LPS-MDL microglial subgroups. One reasonable explanation was that most of the markers in these two subgroups belonged to ISGs [16]. Another possibility was that these two pro-inflammatory microglial subgroups may originate from a common progenitor cellular group, or they were interconvertible under specific stimuli. Both IFN I and LPS immune signaling have been verified to be associated with AD chronic neuroinflammation. But how they initiated microglial activation and neuroinflammation was still largely unknown.

In chronic neuroinflammation, most of the classical pro-inflammatory cytokines (e.g., Ifng, Il1, and Il6) were lowly expressed and were not significantly upregulated in cerebrospinal fluid (CSF), serum, or brain tissue [39,40]. In contrast, the microglial subgroup markers acted as downstream of pro-inflammatory cytokines and were highly expressed and regulated during disease. Therefore, they were reliable biomarkers for neuroinflammation [41]. Transcriptomics marker panels can form regulatory networks that enable disease-specific molecular identification, a strategy already proven effective in certain diagnostic applications [42,43]. In this study, the IFN-MDL constituted a network of antiviral immune responses (Fig 2B). The LPS-MDL was mainly involved in a pro-inflammatory receptor and cytokine signaling network (Fig 2C). The NEUD-MDL constituted a network of growth factor signaling pathways (Fig 2D). And the PROL-MDL was mainly related to TP53-mediated cell cycle pathways (Fig 2E). Together, these four networks represented the major immune regulations in AD microglial activation. Understanding these networks may facilitate the discovery of diagnostic markers or therapeutic targets for AD chronic neuroinflammation.

### Trem2-DAM microglial subgroup may contribute to AD chronic neuroinflammation

**Fig 3:**
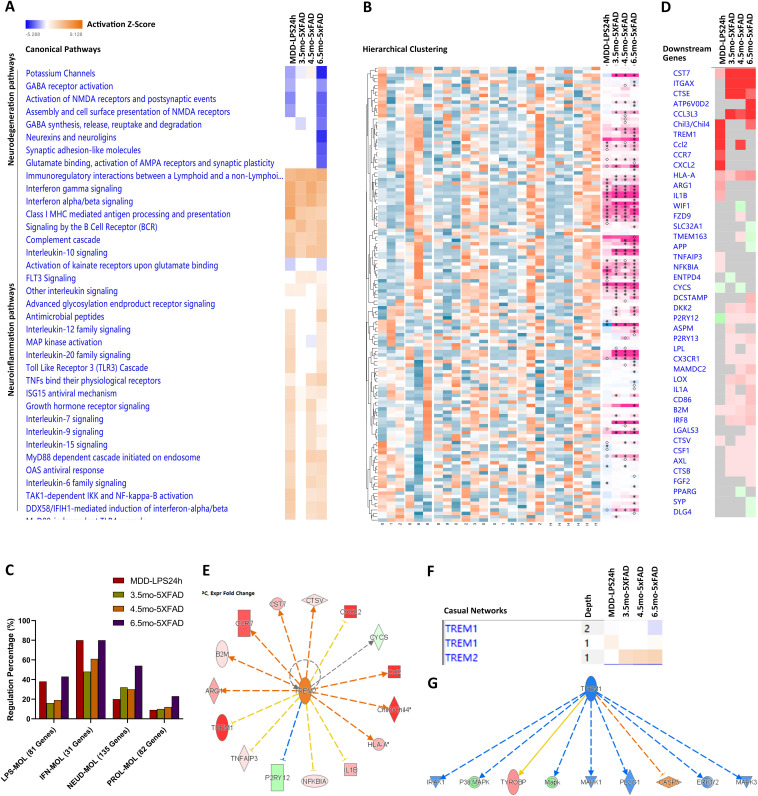
Comparative transcriptomics analysis between MDD acute neuroinflammation and AD chronic neuroinflammation. (A) The IPA Canonical Pathway comparison between the MDD models (MDD-LPS24h) and the AD models (3.5mo-, 4.5mo-, and 6.5mo-5 × FAD) (n = 3). (B) The NEUD-MDL microglial subgroup markers (135 Genes) comparison between the MDD-LPS24h and 5 × FAD models. The significant regulations (p < 0.05) were marked as asterisks (n = 3). (C) The statistical comparison of the regulations of the microglial subgroup markers between the MDD-LPS24h and 5 × FAD models, which was shown in (b) (n = 3). (D) The comparison of the gene regulations in the Trem2 signaling pathway between the MDD-LPS24h and 5 × FAD models (n = 3). (E) The IPA Upstream Regulator analysis revealed the moderate Trem2 signaling activation in the MDD-LPS24h models. (F) The IPA Causal Network analysis revealed the different regulations of the Trem1 and Trem2 signaling activation. (G) The IPA Causal Network analysis revealed that the Trem1 signaling pathway was moderately inhibited by the Trem2 signaling activation in the elder AD models (6.5mo-5 × FAD). Multiple testing corrections were applied in pathway analysis, and the pathways with adjusted p < 0.05 were considered significant. Network robustness was assessed by bootstrap resampling (n = [1000]), and the edges were retained if present in >95% of runs. The IPA Prediction Legend was the same as that in the Fig 2.

To investigate the immune features of the microglial activation in AD, we performed a comparative transcriptomics analysis between MDD acute neuroinflammation and AD chronic neuroinflammation. The IPA Canonical Pathway analysis revealed that the immune activation in the MDD models (MDD-LPS24h) was more pronounced than that in the age-matched AD models (3.5mo-5 × FAD) (Fig 3A). In particular, the pro-inflammatory LPS-MDL and IFN-MDL were more strongly activated in the MDD-LPS24h models compared to the 5 × FAD models (S1A Fig, S1B). In contrast, the anti-inflammatory NEUD-MDL (Trem2-DAM) was markedly elevated in the 5 × FAD models but not in the MDD-LPS24h models (Fig 3B, 3C, S1C Fig). These findings indicate that the robust activation of the anti-inflammatory NEUD-MDL represented a significant feature of AD chronic neuroinflammation.

Notably, several NEUD-MDL markers were induced in both the MDD-LPS24h and the 5 × FAD models, including Cd9, Cd274, Tyrobp, Cd22, Cst7, Tlr2, Ctsz, Spp1, and Cd68 (Fig 3B). However, other NEUD-MDL markers, such as Apoe, Axl, Itgax, Lpl, Plau, Cxcr4, Or5v1b, Lag3, and Dkk2, were specifically upregulated in the 5 × FAD models. Conversely, several major markers of the LPS-MDL (C3, Ccr7, and Tspo) were strongly induced in the MDD-LPS24h models. Additionally, the IFN-MDL markers were highly induced in both the MDD-LPS24h and the 5 × FAD models (S1 Fig B), suggesting that the IFN-MDL may represent a shared microglial subgroup present in both acute and chronic neuroinflammation.

The pro-inflammatory Trem1 signaling and the anti-inflammatory Trem2 signaling were well-established immune signaling pathways involved in neuroinflammation [44]. In our analysis, both the anti-inflammatory Trem2 signaling and the NEUD-MDL (Trem2-DAM) were highly upregulated and progressively increased with age in the 5 × FAD models (Fig 3B-D). In contrast, the Trem2 signaling pathway was only moderately activated in the MDD-LPS24h models (Fig 3E). Remarkably, in the MDD-LPS24h models, the Trem1 gene expression was dramatically upregulated by 39-fold (p < 0.001), accompanied by robust activation of the pro-inflammatory TREM1 signaling pathway. Conversely, the Trem1 signaling pathway was moderately inhibited in the 6.5mo-5 × FAD models (Fig 3F, 3G). This may be attributed to age-associated upregulation of anti-inflammatory TREM2 signaling, which in turn suppressed TREM1-mediated pro-inflammatory responses.

The IPA Upstream Regulator analysis revealed that multiple upstream cytokines were activated in both MDD acute neuroinflammation and AD chronic neuroinflammation. The Top 30 cytokines included several pro-inflammatory factors and growth factors that were typically expressed in activated microglia and reactive astrocytes, such as Ifng, Ifn I, Tnf, Il1b, and Csf2 (S2 Fig A). Thus, cytokines alone may not serve as reliable diagnostic markers to distinguish acute from chronic neuroinflammation. However, the Top 30 gene regulations of the Tlr4 signaling pathway in the MDD-LPS24h models were quite higher than those in the 5 × FAD (S2 Fig B). Many of these genes overlapped with the LPS-MDL markers, further supporting the hypothesis that disease-specific microglial subgroup markers could be diagnostic markers of AD chronic neuroinflammation.

Neuroinflammation impairs neural functions by directly disrupting synaptic organization and structure [45]. In our analysis, the dysregulation of the “Neurotransmitters and Other Nervous System Signaling” pathway was more pronounced in the MDD-LPS24h models than in the age-matched 5 × FAD models (S2 Fig C). Importantly, the major presynaptic marker, the Syp gene (the Synaptophysin gene), and the major postsynaptic marker, the Dlg4 gene (the Psd95 gene), were significantly downregulated in the 6.5mo-5 × FAD models (p < 0.05) (S2 Fig D, E). This synaptic dysregulation was closely correlated with the cognitive disorder and behavioral deficits observed in the 5 × FAD models at this age [46].

Astrocytic activation is another hallmark of neuroinflammation [47]. In this study, we compared the activation levels of microglia and astrocytes based on the Gene Set Enrichment Analysis (GSEA) of the IPA and the Gene Ontology (GO) pathways. In the age-matched 5 × FAD models, the normalized enrichment score (NES) of the “Microglial cell activation” was higher than that of the “Astrocyte activation” (S2 Fig F, G), suggesting that microglial activation may play a more prominent role in AD chronic neuroinflammation.

The above-mentioned microglial modules were defined by meta-analysis of transcriptomics data across multiple neuroinflammation-related diseases, representing common microglial subgroups whose expression profiles vary across diseases. To further characterize their regulation, we first verified the major anti-inflammatory Trem2 pathway by comparing the expression profiles of different microglial modules, and further identified several important regulatory pathways, including the Trem2-Trem1 antagonism and the Trem2-Arg1/B2m regulon. We proposed that this novel data-mining strategy, “from markers to pathways”, may be broadly applicable to the study of complex cellular subgroups.

In summary, our comparative transcriptomics analysis demonstrated that Trem2 signaling drove the formation of a robust anti-inflammatory microglial subgroup, the NEUD-MDL (Trem2-DAM), within the context of AD amyloid pathology. The cellular proportion of the NEUD-MDL (Trem2-DAM) progressively increased with age, competing with the pro-inflammatory microglial activation, and thereby contributing to the development of AD chronic neuroinflammation.

### Microglial activation preceded AD chronic neuroinflammation

Chronic neuroinflammation plays critical roles in AD pathology, contributing to synaptic dysfunction, astrocytic and oligodendrocytic impairments, and neuronal loss. The previous microglia-depletion research has demonstrated that microglial activation was essential for initiating and maintaining neuroinflammation in AD models [48]. Specifically, the functions of activated microglia may vary during aging. Firstly, the early-stage pro-inflammatory microglia secreted cytokines to initiate neuroinflammation signaling cascades [49,50]. Secondly, the proliferation of the activated microglia and the resulting microgliosis further reinforced neuroinflammation [51]. Thirdly, the reactive astrocytes and oligodendrocytes activated by pro-inflammatory microglia exacerbated neuroinflammation [52,53].

**Fig 4:**
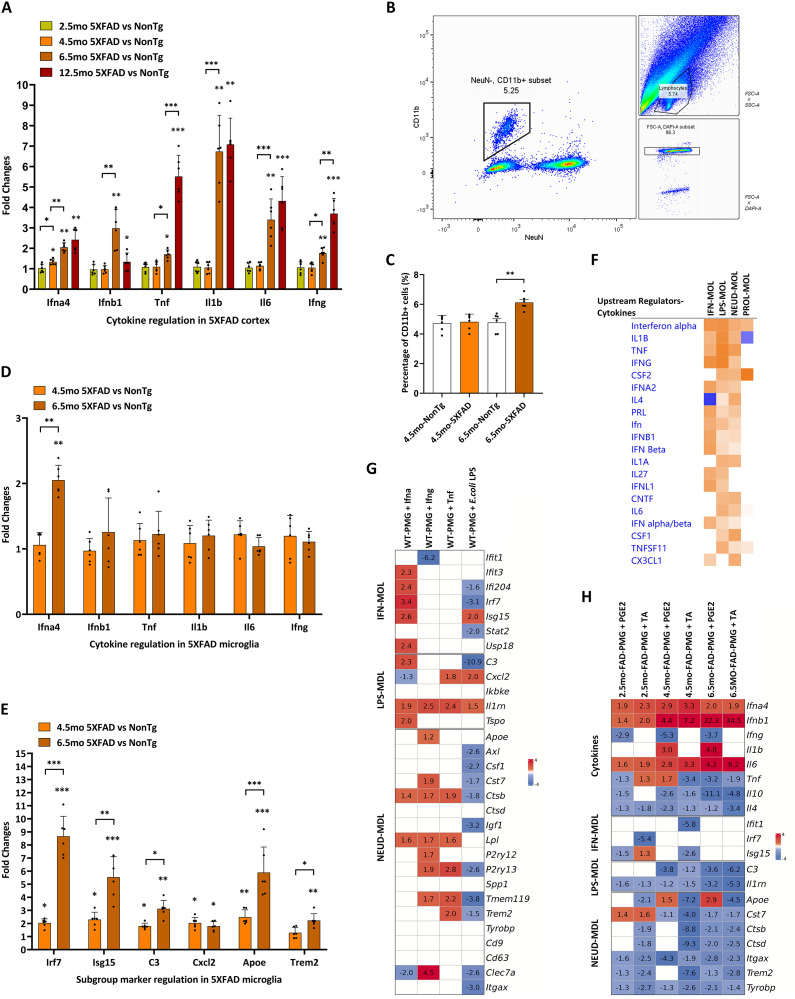
Co-regulation of the pro-inflammatory and anti-inflammatory microglial subgroups in AD chronic neuroinflammation. (A) The AD chronic neuroinflammation gradually increased during aging in the 5 × FAD models. The relative expressions of the major pro-inflammatory cytokines were calculated by qRT-PCR (n = 6). (B) The gating scheme of the Fluorescence-activated cell sorting (FACS) of the CD11b+ brain myeloid cells. (C) The cell number counting in the FACS data revealed microgliosis. The percentage of the CD11b+ cells in the DAPI+ Cells was statistically analyzed (n = 6). (D) The expression levels of the major pro-inflammatory cytokines were not significantly upregulated in the CD11b+ brain myeloid cells of the 4.5mo-5 × FAD and the 6.5mo-5 × FAD models (n = 6). (E) The major microglial subgroup markers were significantly regulated in the CD11b+ brain myeloid cells of the 4.5mo-5 × FAD and the 6.5mo-5 × FAD models (n = 6). (F) The IPA Upstream Regulator analysis predicted the Top 20 upstream regulators of the microglial subgroups. The significance lists were sorted by the Activation Z-Score. (G) The verification of the major upstream regulators of the microglial subgroups in the in vitro cultured 2.5mo-wild-type (WT) primary microglial cells (WT-PMG) by the pharmacological activation assays (n = 3). (H) The verification of the major upstream regulators of the microglial subgroups in the in vitro cultured 5 × FAD primary microglial cells (FAD-PMG) by inhibiting assays (n = 3). Only the fold changes of the significant regulations (p < 0.05) were displayed in the heat map. Multiple testing corrections were applied in pathway analysis, and the pathways with adjusted p < 0.05 were considered significant. The IPA Prediction Legend was the same as that in the Fig 2. The significance levels (p < 0.05, p < 0.01, p < 0.001) were marked as symbols (*, **, ***), respectively.

In this study, the major pro-inflammatory cytokines (e.g., Ifna4, Ifnb1, Tnf, Il1b, Il6, and Ifng) were significantly upregulated in the cortex of the 5 × FAD models, with fold changes progressively increasing during aging (p < 0.05) (Fig 4A). This trend indicated that AD chronic neuroinflammation progressed in parallel with amyloidosis. In addition, the quantity of the microglial cells was significantly increased in the 6.5mo-5 × FAD models (p < 0.01) (Fig 4B, 4C). However, in FACS-purified microglia, the pro-inflammatory cytokines were not significantly altered (Fig 4D). These findings suggested that microglial proliferation and microgliosis, rather than cytokine upregulation, contributed to the elevated cytokine production in AD chronic neuroinflammation. In accord with the scRNAseq data, the major markers of IFN-MDL, LPS-MDL, and NEUD-MDL (Trem2-DAM) were all significantly upregulated in the FACS-purified microglia of the 5 × FAD models (p < 0.05) (Fig 4E). Notably, the upregulation of these microglia subgroup markers started in 4.5mo-5 × FAD FACS-purified microglia (Fig 4E), whereas the upregulation of the pro-inflammatory cytokines started in the 6.5mo-5 × FAD models (Fig 4A). This temporal difference suggested that the cytokine-independent microglial activation preceded the onset of AD chronic neuroinflammation.

In summary, the microglial activation acted as an early driving force of AD chronic neuroinflammation. As an early event, the cytokine-independent microglial activation and microgliosis may induce subsequent alterations in the local microenvironment, such as the release of pro-inflammatory mediators and abnormal angiogenesis, which could represent a shared mechanism between neurodegeneration and neoplastic processes. In addition, as the comparative transcriptomics and FACS-qPCR analysis showed, both the pro-inflammatory microglial subgroups (LPS-MDL and IFN-MDL) and the anti-inflammatory microglial subgroup (NEUD-MDL, Trem2-DAM) were significantly activated at the early stage of AD amyloid pathology. Therefore, we conducted pharmacological activation and inhibition assays on primary microglial culture models to investigate the relationship between the AD microglial subgroups.

### Pro-inflammatory and anti- inflammatory microglial subgroups were co-regulated

The IPA Upstream Regulator analysis predicted that several major pro-inflammatory factors (e.g., Tnf, Ifng, and Il1b) may co-regulate markers of both the anti-inflammatory NEUD-MDL (Trem2-DAM) and the pro-inflammatory LPS-MDL and IFN-MDL subgroups (Fig 4F). In the pharmacological activation assays, the Tnf cytokine 8-hour instantaneous activation significantly upregulated multiple markers of the NEUD-MDL (Trem2-DAM) and the LPS-MDL in the in vitro cultured 2.5mo wild-type (WT) primary microglial cells (WT-PMG) (p < 0.05) (Fig 4G). Meanwhile, in the in vitro cultured 5 × FAD primary microglial cells (FAD-PMG), inhibiting Tnf signaling by TNF-alpha Antagonist III, R-7050 (TA), dramatically downregulated most of the major markers of the NEUD-MDL (Trem2-DAM) and the LPS-MDL. In contrast, the TA treatment only modestly downregulated fewer IFN-MDL markers in the 2.5mo-FAD-PMG and the 4.5mo-FAD-PMG.

A similar age-dependent regulation pattern was observed in the NF-κB inhibition assay. However, compared with the substantial downregulation observed after the TA treatment, the NF-κB inhibition only slightly downregulated the markers of the NEUD-MDL (Trem2-DAM) and the LPS-MDL in the 4.5mo-FAD-PMG (Fig 4H). These results suggested that the Tnf cytokine induced microglial activation not only via the Tnf-NF-κB signaling pathway but also through alternative mechanisms. As TNF was a pleiotropic cytokine involved in the activation of microglia, astrocytes, and oligodendrocytes, Tnf may function as a major early upstream regulator of microglial activation, broadly regulating the scRNAseq markers in the 2.5mo-FAD-PMG.

Moreover, additional in vitro cellular model data supported the IPA Upstream Regulators predictions. In the WT-PMG, the IFN-α cytokine significantly induced the IFN-MDL and promoted the formation of the NEUD-MDL (Trem2-DAM) and the LPS-MDL. In contrast, the IFN-γ cytokine primarily induced the NEUD-MDL (Trem2-DAM) and the LPS-MDL (Fig 4G). Interestingly, the high-dose Escherichia coli (E. coli) LPS only induced the LPS-MDL but inhibited the NEUD-MDL (Trem2-DAM) and IFN-MDL. This may result from high-dose E. coli LPS driving M0 quiescent microglia toward an M1 pro-inflammatory phenotype under in vitro conditions.

In summary, multiple major pro-inflammatory cytokines can co-regulate the microglial subgroups, maintaining a delicate balance between pro-inflammatory and anti-inflammatory microglial activation. This balance may underlie the development of AD chronic neuroinflammation. Importantly, the Trem2-mediated anti-inflammatory microglial subgroup, the NEUD-MDL (Trem2-DAM), was also inhibited in the anti-inflammatory treatment. This finding highlights a potential risk that anti-inflammatory therapies may induce adverse effects by inhibiting the protective Trem2-dependent anti-inflammatory microglial activation.

### Re-evaluating diagnostic biomarkers based on AD microglial subgroups

**Fig 5:**
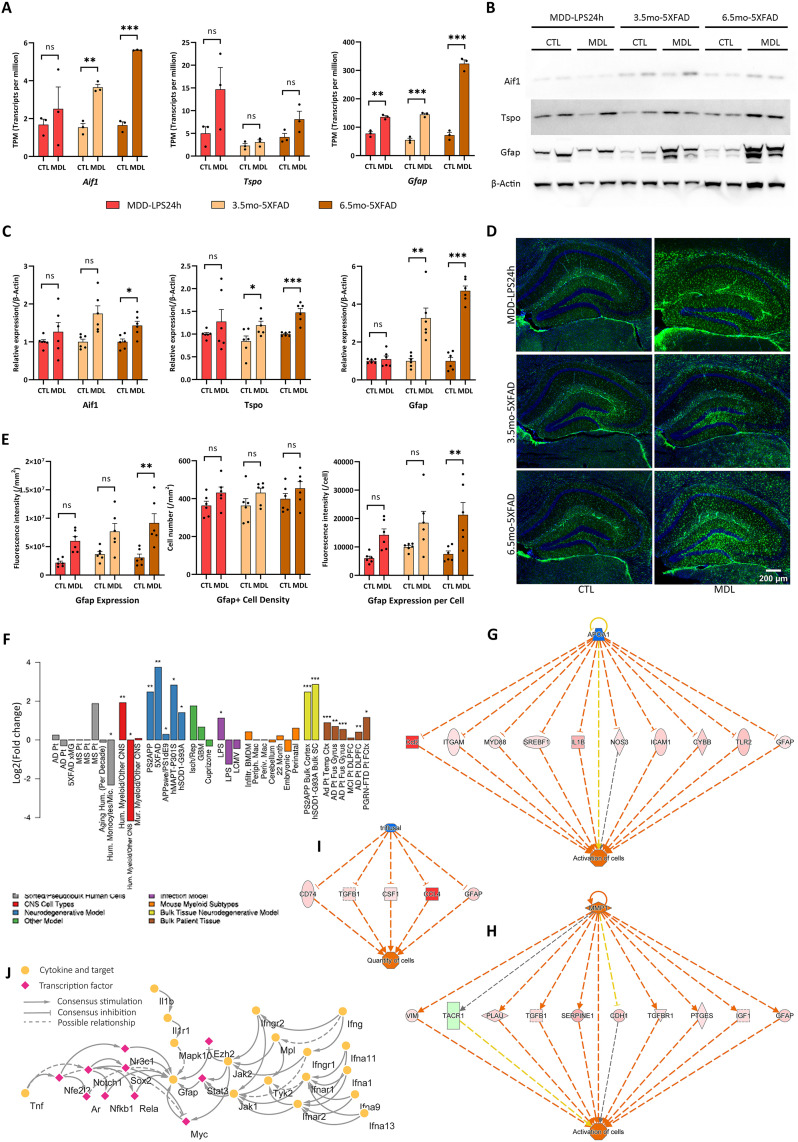
Re-evaluation of the major diagnostic markers of neuroinflammation. (A) The TPM absolute quantification and mRNA expression comparison of *Aif1*, *Tspo*, and *Gfap* based on HPC transcriptomics data (n = 3). (B) The representative figures of the western blotting quantification of Aif1, Tspo, and Gfap in HPC of mouse models. All the full uncropped western blotting images were listed in S1 Raw Images (n = 6). (C) The protein expression comparison of Aif1, Tspo, and Gfap based on western blotting results (n = 6). (D) The representative figures of immunofluorescence staining of Gfap in HPC of mouse models. (E) The protein expression comparison of Aif1, Tspo, and Gfap based on immunofluorescence staining results (n = 6). (F) The regulation profile of the *Gfap* (or *GFAP*) in previously published transcriptomics data. The data was acquired from The Myeloid Landscape 2. (G) The *Gfap* upregulation was involved in the activation of the Regulator Effect Network of “Activation of cells” in MDD-LPS24h models. (H) The *Gfap* upregulation was involved in the activation of the Regulator Effect Network of “Activation of cells” in 5 × FAD models. (I) The *Gfap* upregulation was involved in the activation of the Regulator Effect Network of “Quantity of cells” in 5 × FAD models. (J) The known protein-protein interactions obtained from the OmniPath database revealed that Gfap was positively regulated by pro-inflammatory signaling pathways. CTL = Controls, MDL = Models. Network robustness was assessed by bootstrap resampling (n = [1000]), and the edges were retained if present in >95% of runs. The IPA Prediction Legend was the same as that in the Fig 2. The significance levels (not significantly, *p* < 0.05, *p* < 0.01, *p* < 0.001) were marked as symbols (ns, *, **, ***), respectively.

Comparative transcriptomics analysis provided an opportunity to re-evaluate the diagnostic markers of neuroinflammation. One of the major markers of the LPS-MDL, the 18kD translocator protein (TSPO), has long been used as a biomarker in positron emission tomography (PET) for the diagnosis of AD and other inflammation-related CNS diseases. More recently, the glial fibrillary acidic protein (GFAP), the major marker of the reactive astrocytes, has also been developed as a biomarker for detecting neuroinflammation [47]. In this study, the absolute quantification analysis based on normalized transcripts per million (TPM) from transcriptomics data revealed that, in the hippocampus (HPC) of the mouse models, both the expression levels and the upregulation fold changes of the Tspo gene were significantly lower than those of the Gfap gene (Fig 5A). Notably, the gene expression levels of Tspo and the positive internal control Aif1 (Iba1) were not significantly upregulated in the MDD-LPS24h models. In contrast, Gfap was significantly upregulated in both the MDD-LPS24h (p < 0.01) and the 5 × FAD models (p < 0.001).

We further validated these transcriptomics findings at the protein level. The upregulation fold changes of Gfap were significantly higher than those of Tspo and Aif1 in the HPC of the 5 × FAD models (Fig 5B, 5E). Although the GFAP^+^ cell numbers were not significantly increased, the average Gfap protein level per astrocyte was notably elevated (Fig 5E). Recent scRNAseq data showed that the Tspo gene was highly expressed in microglia and moderately expressed in endothelial cells, fibroblasts, pericytes, and astrocytes, whereas the Gfap gene was highly expressed in astrocytes, and moderately expressed in endothelial cells, fibroblasts, pericytes, and microglia (S3 Fig A, B). Therefore, the constitutive high-expression level (TPM) and significant upregulation of Gfap were likely attributable to the high proportion of astrocytes within the CNS (Fig 5F).

The IPA Regulator Effect Network analysis revealed distinct regulatory mechanisms for Gfap in different models. In the MDD-LPS24h model, Gfap promoted “Activation of cells” in cooperation with multiple pro-inflammatory factors and signaling pathways, including Ccl2, Myd88, Il1b, and Tlr2 (Fig 5G). In contrast, in the 5 × FAD models, Gfap promoted “Activation of cells” through pathways associated with several growth factors, such as Tgfb1, Tgfbr1, and Igf1 (Fig 5H). Moreover, Gfap was involved in promoting “Quantity of cells” in cooperation with Cd74, Tgfb1, Csf1, and Ccl4, but only in the 5 × FAD models (Fig 5I). In addition, Gfap was positively regulated by multiple inflammatory cytokines and transcription factors, including Ifn I, Il1b, Tnf, Stat3, and Nfkb1 (Fig 5J), indicating that it can be induced by pro-inflammatory signaling pathways during neuroinflammation.

By contrast, the functions of Tspo were somewhat different from those of Gfap. In both the MDD-LPS24h and the 5 × FAD models, Tspo acted as an upstream regulator of chemotaxis, including “Cell movement of myeloid cells” and “Migration of cells” (S3 Fig C, D). Like Gfap, Tspo was upregulated in both mouse models and postmortem human samples and was positively regulated by multiple inflammatory cytokines and transcription factors (S3 Fig E, F).

In summary, this comparison between Tspo and Gfap highlighted that Gfap exhibits both higher baseline expression and greater upregulation in pathology, making it a promising and potentially superior biomarker for the diagnosis of AD chronic neuroinflammation (Fig 5F). Although Gfap was a typical marker of astrocytes, recent evidence indicates that it was also expressed in microglia [54,55], and importantly, the highly expressed GFAP protein can be detected in blood and plasma samples [56–58]. In contrast, TSPO was mainly expressed in microglia and can only be detected by PET imaging. Its relatively low expression and modest upregulation may result from the limited cellular proportion of microglia within the CNS. Nevertheless, the early Tspo upregulation likely represents an early diagnostic marker, as microglial activation preceded astrocytic activation in neuroinflammation.

### Reassessing drug targets based on AD microglial subgroups

Given that neuroinflammation drives pathological progression, anti-inflammatory therapy has long been considered a promising strategy for treating AD and other inflammation-associated CNS diseases. Over the past decade, several neuroimmunomodulator and anti-inflammatory drug pipelines have been developed. Some of these drugs specifically targeted the microglial subgroups in AD, including the p38-MAPK inhibitors Neflamapimod [59] and Losmapimod [60], the NF-κB inhibitor NE3107 [61], and the CSF1R inhibitor Masitinib [62]. Based on pathway analysis, inhibition of p38-MAPK or NF-κB was predicted to suppress LPS-MDL, whereas inhibition of CSF1R primarily suppressed PROL-MDL. Consistent with this, the preclinical studies have demonstrated that these chemical compounds significantly reduced microglial activation and neuroinflammation, thereby protecting neurons in disease-relevant mouse models.

However, long-term inhibition of pro-inflammatory signaling pathways during the treatment of AD chronic neuroinflammation may also disrupt normal physiological functions in neurons, astrocytes, and other cells. For example, the NF-κB pathway is essential for maintaining basic neural functions, including synaptic plasticity, learning and memory, synapse to nuclear communication, developmental growth, and neuronal survival [63]. Moreover, the NEUD-MDL (Trem2-DAM) has been recognized as neuroprotective, as it participated in phagocytosing Aβ protein and inhibiting neuroinflammation in AD. Thus, pharmacological activation of the NEUD-MDL (Trem2-DAM) signaling pathway has been proposed as a potential immunotherapeutic method for AD [64]. Nevertheless, as mentioned above, the NEUD-MDL (Trem2-DAM) was also co-regulated by pro-inflammatory cytokines. Consequently, anti-inflammatory treatments may inadvertently suppress the neuroprotective functions of the NEUD-MDL (Trem2-DAM) (Fig 4H).

The molecular property predictions further indicated that most anti-inflammatory chemical compounds possessed the ability to penetrate the blood-brain barrier (BBB), although their efficiencies varied over a wide range (S4 Table). Since the BBB penetration significantly influenced therapeutic efficacy in neurological diseases, the pharmacological immunoassays conducted *in vitro* cellular cultures may not accurately reflect the *in vivo* drug bioavailability. Therefore, complementary pharmacokinetic analyses were essential for compound development. Furthermore, as the neuroimmunomodulators can also influence peripheral immune pathways, enhancing the BBB penetration could improve central efficacy while reducing the required dosages and minimizing peripheral side effects.

In summary, the co-regulation in the CNS immune network revealed in this study (Fig 4F-H) suggested that anti-inflammatory treatments may concurrently inhibit both the pro-inflammatory and the anti-inflammatory microglial activation. These findings highlight the need to re-evaluate current therapeutic targets and strategies for anti-inflammatory interventions in AD, with the goal of selectively modulating neuroprotective microglial responses without impairing beneficial functions.

## Discussion

Pharmacological targeting of neuroinflammation and microglial activation has been considered an effective immunotherapeutic strategy in AD and other inflammation-associated CNS diseases. However, the advantages and potential drawbacks of such approaches remain insufficiently understood due to the limited systemic characterization of neuroinflammatory mechanisms. In recent years, advances in bioinformatics data mining of transcriptomics have revealed a new immune landscape in CNS diseases. In particular, scRNAseq studies have uncovered many previously unrecognized properties of the *in vivo* immune cells. Nevertheless, interpreting and applying these large-scale “Omics Big Data” for diagnostic and drug development purposes continues to present major challenges.

In this study, we established a workflow to investigate the immune regulation networks of the microglial subgroups involved in AD chronic neuroinflammation. Through the bioinformatics data mining and comparative transcriptomics analyses, we found that the upstream regulations of the pro-inflammatory and anti-inflammatory microglial subgroups partially overlapped. The pharmacological activation and inhibition assays in primary cellular models further verified that the anti-inflammatory treatments co-regulated multiple microglial subgroups. Notably, the endogenous anti-inflammatory and neuroprotective NEUD-MDL (Trem2-DAM) subgroup was also suppressed by certain anti-inflammatory drugs. This finding suggested that the potential adverse effects of anti-inflammatory immunotherapy on AD and other CNS diseases warrant closer examination.

Moreover, based on the absolute quantification analysis of transcriptomics data, we re-evaluated several diagnostic markers of neuroinflammation. Although this study has revealed only “the tip of the iceberg” of the complex pathological landscape of AD chronic neuroinflammation, our findings provided an opportunity to re-examine the diagnostic markers and therapeutic targets from the perspectives of systematic biology and network pharmacology.

The main limitations of this study included potential technical biases and the unvalidated biological relevance of certain computational predictions. Notably, this work lacked *in vivo* validation: the key computational findings or candidate mechanisms had not been experimentally confirmed in animal models or other *in vivo* systems. To address these gaps, future studies should incorporate validation in appropriate animal models, apply orthogonal experimental approaches, and integrate longitudinal and multi-cohort multi-omics data. We anticipated that such combined efforts would enable a more accurate assessment of both the therapeutic benefits and potential risks associated with targeting neuroinflammation in AD and related CNS disorders.

## Supporting information

S1 Raw Images The pdf file of the raw images of western blotting experiments. (PDF)

S1 Table The activators and inhibitors used in this study. (DOCX)

S2 Table The murine primers used in this study. (DOCX)

S3 Table The IPA Causal Network analysis of the major AD risk factors. (DOCX)

S4 Table The major predicted molecular properties of the chemical compounds used or discussed in this study. (DOCX)

S5 Table The differentially expressed genes (DEGs) list and the transcripts per million (TPM) list of the comparative transcriptomics analysis. (XLS)

S1 Fig The microglial subgroup markers comparison between the MDD-LPS24h and 5× FAD models. (A) The LPS-MDL comparison revealed that the LPS-MDL induction in the MDD-LPS24h models was more significant than that in the age-matched 3.5mo-5 × FAD models (n = 3). (B) The IFN-MDL comparison revealed that the IFN-MDL induction in the MDD-LPS24h models was more significant than that in the age-matched 3.5mo-5 × FAD models (n = 3). (C) The NEUD-MDL comparison revealed that the NEUD-MDL induction in the 5 × FAD models was more significant than that in the age-matched MDD-LPS24h models (n = 3). (D) The PROL-MDL comparison revealed that the PROL-MDL induction was enhanced during ageing in the 5 × FAD models (n = 3). The significant regulations (p < 0.05) were marked as asterisks (n = 3).
(TIF)

S2 Fig The data mining of the comparative transcriptomics of the MDD-LPS24h models and the 5 × FAD models. (A) The IPA Upstream Regulators analysis revealed that the Top 30 upstream cytokines were involved in both the MDD-LPS24h and the 5× FAD models (n = 3). (B) The comparison of the gene regulations in the Tlr4 signaling pathway between the MDD-LPS24h and 5 × FAD models (n = 3). (C) The comparison of the Neurotransmitters and Other Nervous System Signaling pathways between the MDD-LPS24h and 5 × FAD models (n= 3). (D) The gene downregulation of the presynaptic marker, the *Syp* gene (the Synaptophysin gene), in the 6.5mo-5 × FAD models (n= 3). CTL = Controls, MDL= Models. (E) The gene downregulation of the postsynaptic marker, the *Dlg4* gene (the Psd95 gene), in the 6.5mo-5× FAD models (n= 3). (F) The representative figures of Gene Set Enrichment Analysis (GSEA) of the “Gene Ontology (GO)-Microglia cell activation” pathway (n= 3). (G) The GSEA normalized enrichment score (NES) comparison between the microglial activation and the astrocytic activation (n = 3). CTL = Controls, MDL = Models. Multiple testing corrections were applied in pathway analysis, and the pathways with adjusted *p* < 0.05 were considered significant. The IPA Prediction Legend was the same as that in the Fig 2. The significance levels (not significantly, *p* < 0.05, *p* < 0.01, *p *< 0.001) were marked as symbols (ns, *, **, ***), respectively.
(TIF)

S3 Fig The bioinformatics data for re-evaluating the major diagnostic markers of neuroinflammation. (A) The mean normalized expression of the *Tspo* gene in multiple cells based on scRNAseq data analysis. (B) The mean normalized expression of the *Gfap* gene in multiple cells based on scRNAseq data analysis. (C) The Regulator Effect Network analysis revealed that Tspo was the upstream “Regulator” of the “Cell movement of myeloid cells” in the MDD-LPS24h models (n = 3). (D) The Regulator Effect Network analysis revealed that Tspo was the upstream “Regulator” of the “Migration of cells” in the 5 × FAD models (n = 3). (E) The regulation profile of the Tspo (or TSPO) in previously published transcriptomics data. The data were acquired from The Myeloid Landscape 2. (F) The known protein-protein interactions obtained from the OmniPath database revealed that Tspo was positively regulated by pro-inflammatory signaling pathways. Network robustness was assessed by bootstrap resampling (n = [1000]), and the edges were retained if present in >95% of runs. The IPA Prediction Legend was the same as that in the Fig 2. The significance levels (*p *< 0.05, *p *< 0.01, *p *< 0.001) were marked as symbols (*, **, ***), respectively.
(TIF)
