# Supplementary material for: Co-regulation of microglial subgroups in Alzheimer’s amyloid pathology: Implications for diagnosis and drug development
Source: PLoS One. 2025 Dec 5;20(12):e0337741. doi: 10.1371/journal.pone.0337741 (PMC12680192; doi:10.1371/journal.pone.0337741)

The full uncropped western blotting images

Co-regulation of microglial subgroups in Alzheimer's amyloid pathology: Implications for diagnosis and drug development

Yu Zhou[1], Yukuan Huang[1], Yangchang Fan[1], Feng Xue[1*]
[1] Hwamei College of Life and Health Sciences, Zhejiang Wanli University, Ningbo, Zhejiang, China
[*] Corresponding author
E-mail: xuefeng@zwu.edu.cn (FX)

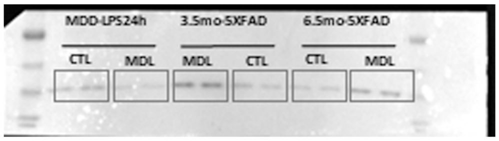

Note: The sample loading order in each membrane, the figure legends were same as those in the Figure 5.

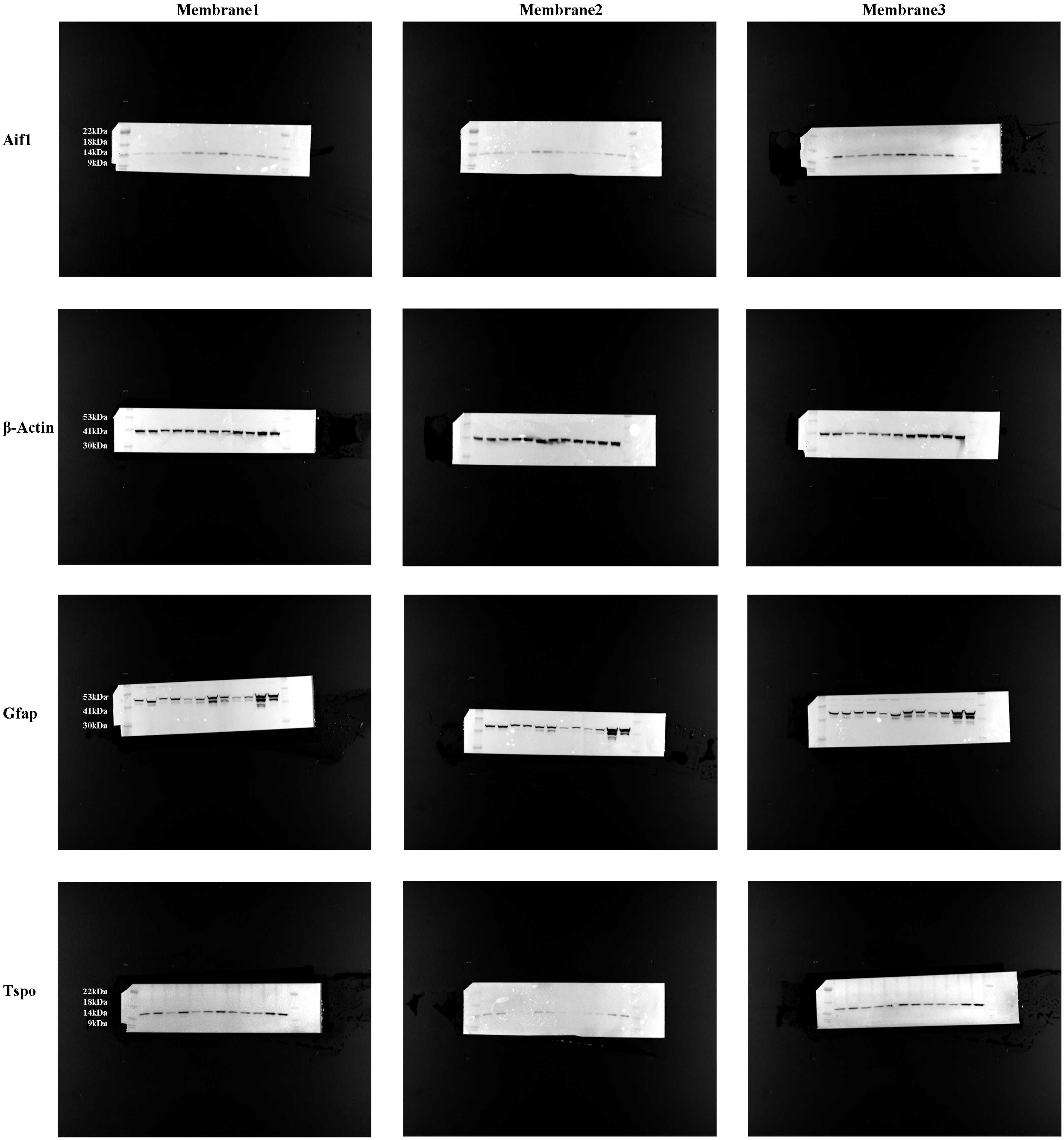

Supplement: S1 Raw Images — (PDF) [file pone.0337741.s001.pdf]
