# Supplementary material for: Co-regulation of microglial subgroups in Alzheimer’s amyloid pathology: Implications for diagnosis and drug development
Source: PLoS One. 2025 Dec 5;20(12):e0337741. doi: 10.1371/journal.pone.0337741 (PMC12680192; doi:10.1371/journal.pone.0337741)
Supplement: S1 Table — (DOCX) [file pone.0337741.s002.docx]

**Table S1.** **The activators and inhibitors used in this study.**

| Name | Company | Dose |
| --- | --- | --- |
| Recombinant Mouse IFN-α (carrier-free) | BioLegend (752802) | 10ng/mL |
| Recombinant Mouse IFN-γ (carrier-free) | BioLegend (575302) | 10ng/mL |
| Tumor Necrosis Factor-α human | Sigma (H8916-10UG) | 10ng/mL |
| Standard LPS, *E. coli* K12 | InvivoGen (tlrl-eklps) | 10ng/mL |
| TNF-alpha Antagonist III, R-7050 | EMD Millipore (654257-10MG) | 5uM |
| Prostaglandin E2, EP1, EP2, EP3, and EP4 ligand (PGE2) | Abcam (ab144539) | 100uM |
