# Supplementary material for: Co-regulation of microglial subgroups in Alzheimer’s amyloid pathology: Implications for diagnosis and drug development
Source: PLoS One. 2025 Dec 5;20(12):e0337741. doi: 10.1371/journal.pone.0337741 (PMC12680192; doi:10.1371/journal.pone.0337741)
Supplement: S2 Table — (DOCX) [file pone.0337741.s003.docx]

**Table S2.** **The murine primers used in this study.**

| Gene name | Forward primer | Reverse primer |
| --- | --- | --- |
| mIfna4 | CTTTCCTCATGATCCTGGTAATGAT | AATCCAAAATCCTTCCTGTCCTTC |
| mIfnb1 | CCCTATGGAGATGACGGAGA | CCCAGTGCTGGAGAAATTGT |
| mIrf7 | CAATTCAGGGGATCCAGTTG | AGCATTGCTGAGGCTCACTT |
| mIsg15 | CTAGAGCTAGAGCCTGCAG | AGTTAGTCACGGACACCAG |
| mTnf | CAGGCGGTGCCTATGTCTC | CGATCACCCCGAAGTTCAGTAG |
| mIl1b | GAAATGCCACCTTTTGACAGTG | TGGATGCTCTCATCAGGACAG |
| mIl6 | TAGTCCTTCCTACCCCAATTTCC | TTGGTCCTTAGCCACTCCTTC |
| mC3 | CCAGCTCCCCATTAGCTCTG | GCACTTGCCTCTTTAGGAAGTC |
| mCXCL2 | CCAACCACCAGGCTACAGG | GCGTCACACTCAAGCTCTG |
| mCst7 | GCCGAACTACATGCAGGAAGA | GGCAGGGTTGGTTTGGAAGT |
| mCtsb | TCCTTGATCCTTCTTTCTTGCC | ACAGTGCCACACAGCTTCTTC |
| mCtsd | GCTTCCGGTCTTTGACAACCT | CACCAAGCATTAGTTCTCCTCC |
| mCd63 | GAAGCAGGCCATTACCCATGA | TGACTTCACCTGGTCTCTAAACA |
| mTrem2 | CTGGAACCGTCACCATCACTC | CGAAACTCGATGACTCCTCGG |
| mIfit1 | CTGAGATGTCACTTCACATGGAA | GTGCATCCCCAATGGGTTCT |
| mIfng | ATGAACGCTACACACTGCATC | CCATCCTTTTGCCAGTTCCTC |
| mItgax | CTGGATAGCCTTTCTTCTGCTG | GCACACTGTGTCCGAACTCA |
| mTmem119 | CCTACTCTGTGTCACTCCCG | CACGTACTGCCGGAAGAAATC |
| mTyrobp | GAGTGACACTTTCCCAAGATGC | CCTTGACCTCGGGAGACCA |
| mGapdh | GACTTCAACAGCAACTCCCAC | TCCACCACCCTGTTGCTGTA |
| mIfit3 | TCAGGCTTACGTTGACAAGGT | CACACTTTAGGCGTGTCCATC |
| mIfi204 | GAGCAAGGCGGCTAAGGAA | GCTGTGGAGTATTGGTGACTG |
| mStat2 | TCCTGCCAATGGACGTTCG | GTCCCACTGGTTCAGTTGGT |
| mUsp18 | TTGGGCTCCTGAGGAAACC | CGATGTTGTGTAAACCAACCAGA |
| mIkbke | ACCACTAACTACCTGTGGCAT | CCTCCCCGGATTTCTTGTTTC |
| mIl1rn | GCTCATTGCTGGGTACTTACAA | CCAGACTTGGCACAAGACAGG |
| mTspo | GCCTACTTTGTACGTGGCGAG | ATGGCTGAATACAGTGTTGCC |
| mApoe | CTGACAGGATGCCTAGCCG | CGCAGGTAATCCCAGAAGC |
| mAxl | TGGTGAGGGAGGAGCATGTT | AAAAGAAGGGGAGCTTGCTGA |
| mCsf1 | GGCTTGGCTTGGGATGATTCT | GAGGGTCTGGCAGGTACTC |
| mIgf1 | CTGGACCAGAGACCCTTTGC | GGACGGGGACTTCTGAGTCTT |
| mLpl | GGGAGTTTGGCTCCAGAGTTT | TGTGTCTTCAGGGGTCCTTAG |
| mP2ry12 | ATGGATATGCCTGGTGTCAACA | AGCAATGGGAAGAGAACCTGG |
| mP2ry13 | ATGCTCGGGACAATCAACACC | GATGTGGACGAACACCCAGAG |
| mSpp1 | AGCAAGAAACTCTTCCAAGCAA | GTGAGATTCGTCAGATTCATCCG |
| mCd9 | ATGCCGGTCAAAGGAGGTAG | GCCATAGTCCAATAGCAAGCA |
| mClec7a | GACTTCAGCACTCAAGACATCC | TTGTGTCGCCAAAATGCTAGG |
