# Supplementary material for: Co-regulation of microglial subgroups in Alzheimer’s amyloid pathology: Implications for diagnosis and drug development
Source: PLoS One. 2025 Dec 5;20(12):e0337741. doi: 10.1371/journal.pone.0337741 (PMC12680192; doi:10.1371/journal.pone.0337741)
Supplement: S3 Table — (DOCX) [file pone.0337741.s004.docx]

**Table S3.** **The IPA-Causal Network analysis of the major AD risk factors.**

| Master Regulator | Molecule Type | Participating Regulators | P-value of overlap | Target Molecules in Dataset |
| --- | --- | --- | --- | --- |
| TLR3 | transmembrane receptor | IKBKB, IRF3, JUN, MAPK14, MYD88, NFkB (complex), NFKB1, NFKBIA, RELA, STAT1, TLR3 | 1.51E-05 | APOE, CLU, HLA-DRB5, INPP5D, MEF2C, PICALM, PLCG2, PTK2B, SORL1, SPI1 |
| ATPase | group | Akt, ATPase, DDX3X, DHX9, E2F1, HSP90AA1, HSPA5, HSPD1, MYC, NFKB1, RELA, SMAD2, SMARCA4, SMARCA5, STAT1, STAT3, VCP | 2.52E-05 | ABCA7, BIN1, CD2AP, CD33, CLU, EPHA1, HLA-DRB5, IL1RAP, INPP5D, MEF2C, PLCG2, SPI1 |
| MAPK13 | kinase | Ap1, CCND3, CEBPA, CXCL8, DDIT3, E2F1, ERK1/2, FOS, GATA2, IL10, IL1B, JUN, KAT5, MAPK13, MEF2C, mir-21, MYC, NFAT5, NFE2L2, NFKBIA, PGR, PRKAA1, PTK2, SP3, STAT3 | 2.84E-05 | ABCA7, APOE, BIN1, CD2AP, CLU, CR1, EPHA1, HLA-DRB5, MEF2C, NME8, PICALM, PTK2B, SORL1, SPI1, TREM2 |
| Notch | group | Akt, AKT1, E2F1, GATA2, IL10, IRF3, KRAS, LCK, Mapk, MAPK14, MAPK8, MYC, MYOD1, Notch, NOTCH1, PGR, PI3K (complex), TP73 | 4.56E-05 | ABCA7, APOE, BIN1, CD2AP, CD33, CLU, CR1, EPHA1, HLA-DRB1, NME8, PLCG2, SORL1, TREM2 |
| IKBKG | kinase | IKBKB, IKBKG, IRF3, JUN, MAP2K4, NFkB (complex), NFKB1, NFKBIA, RELA, STAT1, ZAP70 | 6.09E-05 | APOE, CLU, HLA-DRB5, MEF2C, PICALM, PLCG2, PTK2B, SORL1, SPI1 |
| CXCL16 | cytokine | Akt, CEBPA, CSF2, CXCL16, CXCL8, IL10, IL1B, IRF3, KRAS, MYC, NFkB (complex), NFKBIA, PI3K (complex), TP53 | 6.97E-05 | ABCA7, APOE, BIN1, CD33, CLU, CR1, FERMT2, HLA-DRB1, IL1RAP, MEF2C, PICALM, PLCG2, SORL1, SPI1, TREM2 |
| TBK1 | kinase | Akt, AKT1, IKBKB, IRF3, JUN, NFkB (complex), NFKBIA, RELA, STAT1, TBK1 | 7.26E-05 | APOE, CD33, CLU, HLA-DRB5, PICALM, PLCG2, PTK2B, SORL1, SPI1 |
| PI3K (family) | group | Akt, IRF3, MYC, NFKB1, NFKBIA, Notch, PI3K (family), PPARGC1A, PTK2, SREBF1, STAT1, STAT3, SYK, TP53 | 8.84E-05 | ABCA7, APOE, BIN1, CD33, FERMT2, HLA-DRB5, IL1RAP, MEF2C, PICALM, PLCG2, PTK2B, SORL1 |
| VEGFC | growth factor | AKT1, CEBPA, ERK, ERK1/2, FAS, FLT4, IL10, IRF3, ITGB1, Jnk, KDR, KRAS, MYC, MYCN, PGR, PI3K (complex), PTK2B, SIRT1, SMAD2, SMAD3, SNCA, SP3, STAT1, STAT3, SYK, TNF, VEGFC | 1.16E-04 | ABCA7, BIN1, CD2AP, CLU, CR1, EPHA1, HLA-DRB1, HLA-DRB5, IL1RAP, INPP5D, MEF2C, PLCG2, SPI1, TREM2 |
| IGF2 | growth factor | AGT, Akt, AKT1, CEBPA, CHUK, CXCL8, E2F1, EGFR, ERK, ERK1/2, GATA2, IGF1R, IGF2, IKBKB, IL10, IRF3, Jnk, JUN, KRAS, MAP3K14, Mapk, MAPK1, MAPK14, MTOR, MYC, MYCN, MYOD1, NFkB (complex), NOS2, P38 MAPK, p70 S6k, PGR, PRKCB, SP3, SRC, STAT3, SYK, ZAP70, ZEB1 | 1.60E-04 | ABCA7, APOE, BIN1, CD2AP, CD33, CLU, CR1, EPHA1, HLA-DRB1, HLA-DRB5, MEF2C, NME8, PLCG2, PTK2B, SPI1 |
| CCL1 | cytokine | Akt, CCL1, CEBPA, ERK1/2, IL10, IRF3, KRAS, MYC, PGR, PI3K (complex), SMAD2, SMAD3, SP3, SRF, STAT1, STAT3, TP53 | 1.61E-04 | ABCA7, BIN1, CD2AP, CD33, CR1, EPHA1, FERMT2, HLA-DRB1, HLA-DRB5, IL1RAP, INPP5D, MEF2C, PLCG2, SPI1 |
| TNFSF8 | cytokine | Akt, CEBPA, ERK1/2, IL10, IL1B, Inflammasome, MYC, NFKBIA, PGR, PRKAA1, SMAD2, SMAD3, SP3, SRF, STAT3, TNFSF8, TP53 | 1.98E-04 | ABCA7, BIN1, CD2AP, CD33, CR1, EPHA1, FERMT2, HLA-DRB5, IL1RAP, INPP5D, PICALM, PTK2B, SORL1, TREM2 |
| TH2 Cytokine | group | IL10, IL13, IL4, IL6, TH2 Cytokine | 2.09E-04 | APOE, CD33, CLU, CR1, HLA-DRB5, INPP5D, SPI1, TREM2 |
| IFNL3 | cytokine | Akt, CEBPA, ERK1/2, IFNG, IFNL3, IL10, IRF3, JAK1, MYC, NFKBIA, PGR, PRKAA1, SP3, STAT1, STAT3, STAT4, STAT5a/b, STK11, SYK | 3.10E-04 | ABCA7, BIN1, CD2AP, CD33, CLU, CR1, EPHA1, HLA-DRB1, HLA-DRB5, MEF2C, PICALM, PLCG2, SORL1, SPI1 |
| IFNL1 | cytokine | Akt, CEBPA, ERK1/2, IFNG, IFNL1, IL10, IRF3, JAK1, MYC, NFKBIA, PGR, PRKAA1, SP3, STAT1, STAT3, STAT4, STAT5a/b, STK11, SYK | 3.31E-04 | ABCA7, BIN1, CD2AP, CD33, CLU, CR1, EPHA1, HLA-DRB1, HLA-DRB5, MEF2C, PICALM, PLCG2, SORL1, SPI1 |
| B2M | transmembrane receptor | B2M | 4.14E-04 | HLA-DRB5, MEF2C |
| CSF | group | CSF, CSF1, CSF1R, CSF2, CSF3 | 5.85E-04 | APOE, CD33, HLA-DRB1, PTK2B, SPI1 |
| IL9R | transmembrane receptor | IL9R, STAT1, STAT3, STAT5A, STAT5a/b | 9.06E-04 | APOE, CLU, HLA-DRB5, MEF2C, SPI1 |
| IFNGR | complex | Akt, CD274, E2F1, ERBB2, GSK3B, IFNGR, IL4, IRF1, JAK1, JAK2, MYC, NFKBIA, PGR, Rap1, SMAD3, SRF, STAT1, STAT6, SYK, TP53 | 9.85E-04 | ABCA7, APOE, BIN1, CD2AP, CD33, EPHA1, FERMT2, HLA-DRB5, IL1RAP, INPP5D, PICALM, PLCG2, SORL1 |
| JAK1/2 | group | JAK1/2 | 2.84E-03 | HLA-DRB5, PTK2B |
| CSF2RA | transmembrane receptor | Akt, CSF2RA, GATA2, Mapk, MAPK1, MAPK3, MYC, NFKBIA, PGR, PRKAA1, SMAD2, SMAD3, STK11, TP73 | 3.56E-03 | ABCA7, BIN1, CD33, EPHA1, INPP5D, NME8, PICALM, SORL1, TREM2 |
| STAT5a/b | group | STAT5a/b | 4.82E-03 | CLU, MEF2C |
| mir-155 | microRNA | mir-155 | 7.53E-03 | INPP5D, SPI1 |
| TLR2/3/4/9 | group | TLR2/3/4/9 | 1.22E-02 | TREM2 |

**Note:** The ‘Master Regulator’ was the predicted upstream regulator in a Casual Network. The ‘Participating Regulators’ were the predicted midstream regulators in a Casual Network. The ‘Target Molecules in Dataset’ were the downstream functional genes in a Casual Network. The major AD risk factors were mainly enriched in the ‘Target Molecules in Dataset’.
