# Supplementary material for: Co-regulation of microglial subgroups in Alzheimer’s amyloid pathology: Implications for diagnosis and drug development
Source: PLoS One. 2025 Dec 5;20(12):e0337741. doi: 10.1371/journal.pone.0337741 (PMC12680192; doi:10.1371/journal.pone.0337741)
Supplement: S4 Table — (DOCX) [file pone.0337741.s005.docx]

**Table S4. The major predicted molecular properties of the chemical compounds used or discussed in this study.**

|  | **Distribution** | | **Absorption** | | | **Toxicity** | | | | | | | | |
| --- | --- | --- | --- | --- | --- | --- | --- | --- | --- | --- | --- | --- | --- | --- |
| **Compound Name** | BBB Penetration | PPB | Caco-2 Permeability | MDCK Permeability | HIA | AMES Toxicity | Carcinogencity | DILI | H-HT | hERG Blockers | Acute Toxicity Rule | Aquatic Toxicity Rule | Genotoxic Carcinogenicity Rule | NonGenotoxic Carcinogenicity Rule |
| **Neflamapimod** | 0.217 | 1.011 | 0.726 | 1.39E-05 | 0.995 | 0.103 | 0.333 | 0.527 | 0.875 | 0.801 | 0 | 1 | 0 | 0 |
| **NE3107** | 0.132 | 0.898 | 0.711 | -2.78E-06 | 0.989 | 0.416 | 0.705 | 0.528 | 0.962 | 0.021 | 0 | 0 | 0 | 0 |
| **Losmapimod** | 0.2473 | 0.838 | 1.132 | 1.08E-05 | 0.999 | 0.367 | 0.148 | 0.559 | 0.786 | 0.977 | 0 | 1 | 0 | 1 |
| **Masitinib** | 0.016 | 0.895 | 0.471 | 9.36E-07 | 0.976 | 0.027 | 0.219 | 0.546 | 0.974 | 0.994 | 0 | 0 | 1 | 0 |
| **TNF-alpha Antagonist III, R-7050** | 0.881 | 1.093 | 0.934 | 1.04E-05 | 0.994 | 0.016 | 0.458 | 0.538 | 0.907 | 0.298 | 0 | 3 | 3 | 2 |
| **Prostaglandin E2, EP1, EP2, EP3, and EP4 ligand (PGE2)** | 0.066 | 0.939 | 0.168 | -9.97E-06 | 0.983 | 0.012 | 0.367 | 0.531 | 0.921 | 0.042 | 0 | 0 | 0 | 0 |

**Note:** The properties were predicted using HUAWEI Cloud Pangu Drug Molecular Model (http://www.pangu-drug.com/). (1) BBB Penetration (Range value: 0-1): Drugs cross the blood–brain barrier (BBB) to reach their CNS molecular target. The output value is the probability of being BBB+, within the range of 0 to 1. The probability increases as the value increases. (2) PPB: Plasma protein binding. A compound is considered to have a proper PPB if it has predicted value < 90%. (3) Caco-2 Permeability (Log cm/s): The human colorectal adenocarcinoma cell line (Caco-2) was used to estimate drug permeability. Empirical decision: > -5.15: excellent (green); otherwise: poor (red). (4) MDCK Permeability (cm/s): Madin−Darby Canine Kidney cells (MDCK) have been developed as an *in vitro* model for permeability screening. Empirical decision: >2 x 10-6cm/s: excellent (green), otherwise: poor (red). (5) HIA (Range value: 0-1): Human intestinal absorption. Empirical decision: 0-0.3: excellent (green); 0.3-0.7: medium (yellow); 0.7-1.0(++): poor (red). (6) AMES Toxicity (Range value: 0-1): The Ames test for mutagenicity. Empirical decision: 0-0.3: excellent (green); 0.3-0.7: medium (yellow); 0.7-1.0(++): poor (red). (7) Carcinogencity: The carcinogenic mechanism of chemicals may be due to their ability to damage the genome or disrupt cellular metabolic processes. Empirical decision: 0-0.3: excellent (green); 0.3-0.7: medium (yellow); 0.7-1.0(++): poor (red). (8) DILI: Drug-induced liver injury. Empirical decision: 0-0.3: excellent (green); 0.3-0.7: medium (yellow); 0.7-1.0(++): poor (red). (9) H-HT: The human hepatotoxicity. Empirical decision: 0-0.3: excellent (green); 0.3-0.7: medium (yellow); 0.7-1.0(++): poor (red). (10) hERG Blockers: hERG is the human ether-a-go-go related gene. The hERG blockade may lead to palpitations, fainting, or even sudden death. Empirical decision: 0-0.3: excellent (green); 0.3-0.7: medium (yellow); 0.7-1.0(++): poor (red). (11) Acute Toxicity Rule: Molecules containing these substructures may cause acute toxicity during oral administration. There are 20 substructures in this endpoint. (12) Aquatic Toxicity Rule: Molecules containing these substructures may cause toxicity to liquid(water). There are 99 substructures in this endpoint. (13) Molecules containing these substructures may cause carcinogenicity or mutagenicity through genotoxic mechanisms. There are 117 substructures in this endpoint. (14) NonGenotoxic Carcinogenicity Rule: Molecules containing these substructures may cause carcinogenicity through nongenotoxic mechanisms. There are 23 substructures in this endpoint.
