# Supplementary figures and images for: Co-regulation of microglial subgroups in Alzheimer’s amyloid pathology: Implications for diagnosis and drug development
Source: PLoS One. 2025 Dec 5;20(12):e0337741. doi: 10.1371/journal.pone.0337741 (PMC12680192; doi:10.1371/journal.pone.0337741)

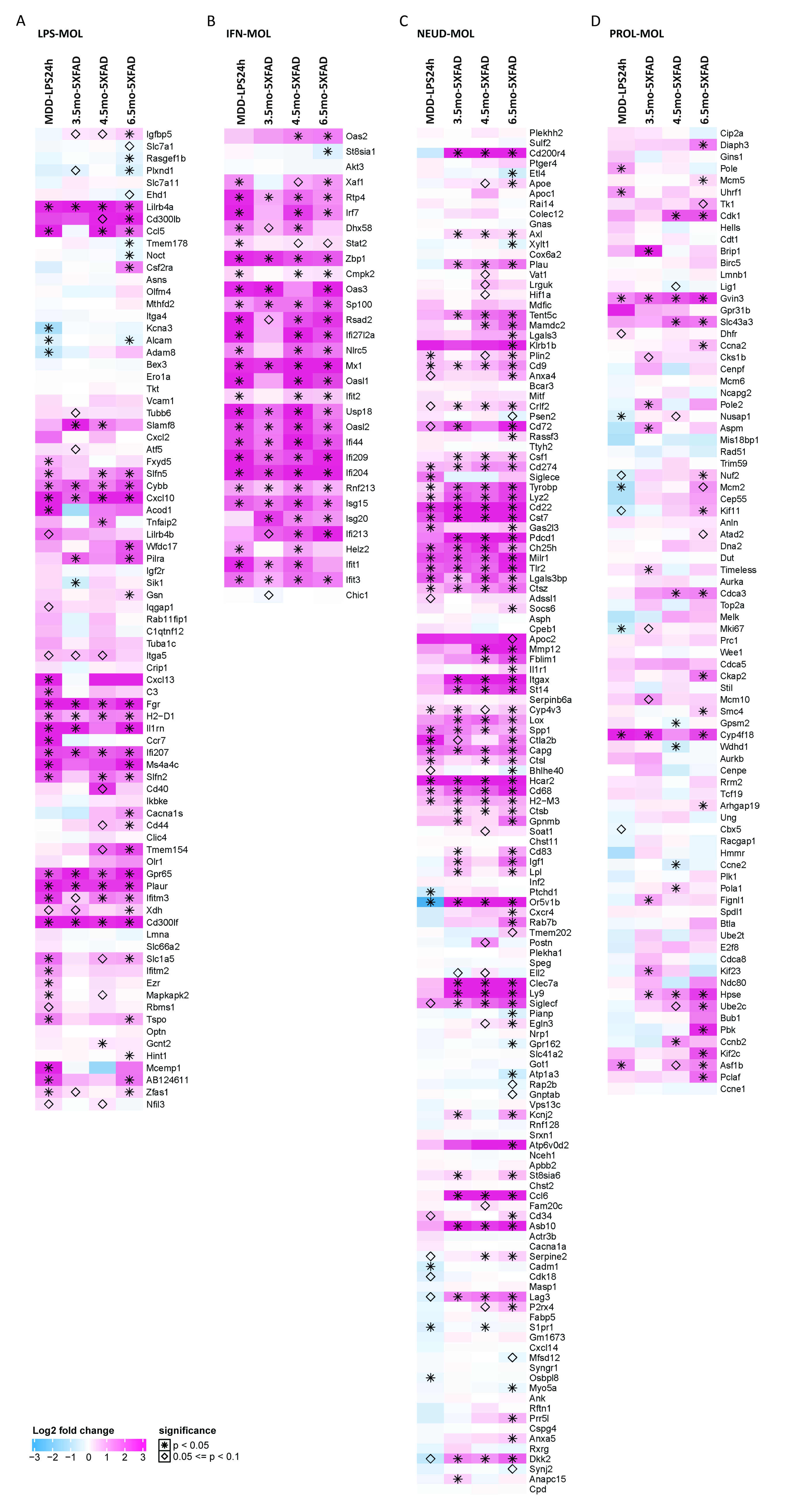

Supplement: S1 Fig — (A) The LPS-MDL comparison revealed that the LPS-MDL induction in the MDD-LPS24h models was more significant than that in the age-matched 3.5mo-5 × FAD models (n = 3). (B) The IFN-MDL comparison revealed that the IFN-MDL induction in the MDD-LPS24h models was more significant than that in the age-matched 3.5mo-5 × FAD models (n = 3). (C) The NEUD-MDL comparison revealed that the NEUD-MDL induction in the 5 × FAD models was more significant than that in the age-matched MDD-LPS24h models (n = 3). (D) The PROL-MDL comparison revealed that the PROL-MDL induction was enhanced during ageing in the 5 × FAD models (n = 3). The significant regulations (p < 0.05) were marked as asterisks (n = 3). (TIF) [file pone.0337741.s007.tif]

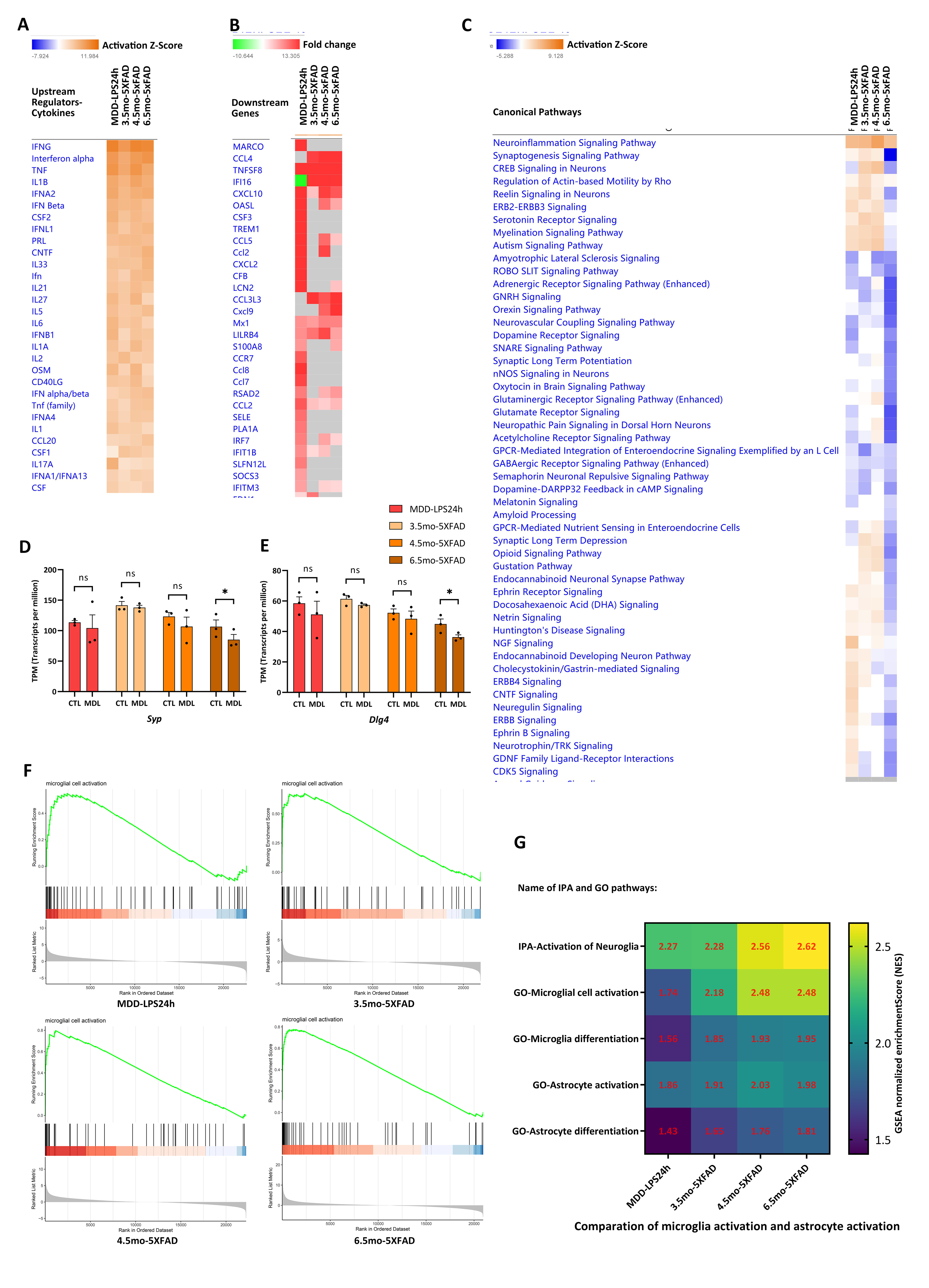

Supplement: S2 Fig — (A) The IPA Upstream Regulators analysis revealed that the Top 30 upstream cytokines were involved in both the MDD-LPS24h and the 5× FAD models (n = 3). (B) The comparison of the gene regulations in the Tlr4 signaling pathway between the MDD-LPS24h and 5 × FAD models (n = 3). (C) The comparison of the Neurotransmitters and Other Nervous System Signaling pathways between the MDD-LPS24h and 5 × FAD models (n= 3). (D) The gene downregulation of the presynaptic marker, the Syp gene (the Synaptophysin gene), in the 6.5mo-5 × FAD models (n= 3). CTL = Controls, MDL= Models. (E) The gene downregulation of the postsynaptic marker, the Dlg4 gene (the Psd95 gene), in the 6.5mo-5× FAD models (n= 3). (F) The representative figures of Gene Set Enrichment Analysis (GSEA) of the “Gene Ontology (GO)-Microglia cell activation” pathway (n= 3). (G) The GSEA normalized enrichment score (NES) comparison between the microglial activation and the astrocytic activation (n = 3). CTL = Controls, MDL = Models. Multiple testing corrections were applied in pathway analysis, and the pathways with adjusted p < 0.05 were considered significant. The IPA Prediction Legend was the same as that in the Fig 2. The significance levels (not significantly, p < 0.05, p < 0.01, p < 0.001) were marked as symbols (ns, *, **, ***), respectively. (TIF) [file pone.0337741.s008.tif]

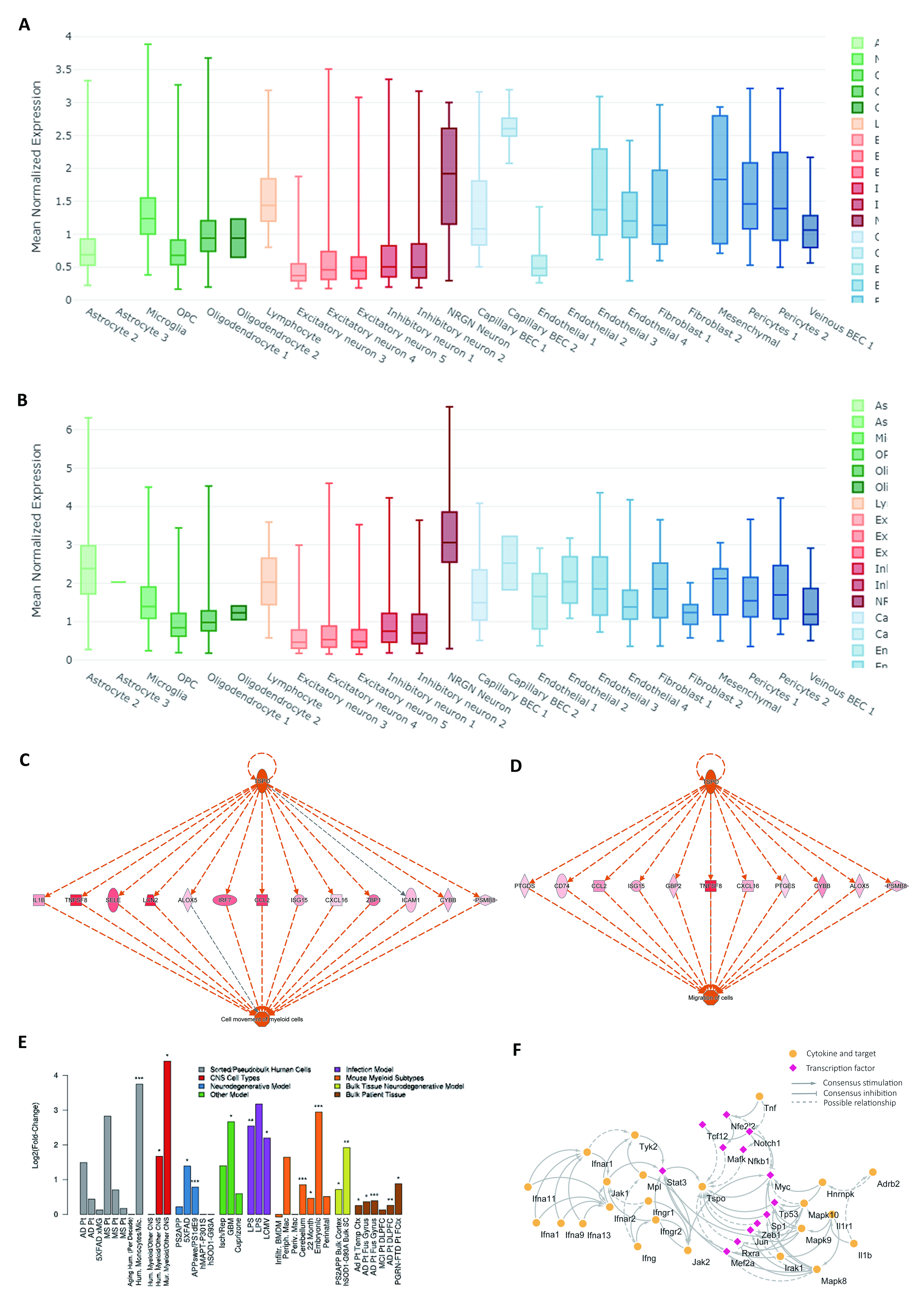

Supplement: S3 Fig — (A) The mean normalized expression of the Tspo gene in multiple cells based on scRNAseq data analysis. (B) The mean normalized expression of the Gfap gene in multiple cells based on scRNAseq data analysis. (C) The Regulator Effect Network analysis revealed that Tspo was the upstream “Regulator” of the “Cell movement of myeloid cells” in the MDD-LPS24h models (n = 3). (D) The Regulator Effect Network analysis revealed that Tspo was the upstream “Regulator” of the “Migration of cells” in the 5 × FAD models (n = 3). (E) The regulation profile of the Tspo (or TSPO) in previously published transcriptomics data. The data were acquired from The Myeloid Landscape 2. (F) The known protein-protein interactions obtained from the OmniPath database revealed that Tspo was positively regulated by pro-inflammatory signaling pathways. Network robustness was assessed by bootstrap resampling (n = [1000]), and the edges were retained if present in >95% of runs. The IPA Prediction Legend was the same as that in the Fig 2. The significance levels (p < 0.05, p < 0.01, p < 0.001) were marked as symbols (*, **, ***), respectively. (TIF) [file pone.0337741.s009.tif]
